# Risk factors for poor outcomes in hospitalised COVID-19 patients: A systematic review and meta-analysis

**DOI:** 10.7189/jogh.11.10001

**Published:** 2021-03-01

**Authors:** You Li, Thulani Ashcroft, Alexandria Chung, Izzie Dighero, Marshall Dozier, Margaret Horne, Emilie McSwiggan, Azwa Shamsuddin, Harish Nair

**Affiliations:** 1 Usher Institute, University of Edinburgh, Edinburgh, UK; 2 Information Services, University of Edinburgh, Edinburgh, UK; 3 Centre for Clinical Brain Sciences, University of Edinburgh, Edinburgh, UK

## Abstract

**Background:**

Understanding the risk factors for poor outcomes among COVID-19 patients could help identify vulnerable populations who would need prioritisation in prevention and treatment for COVID-19. We aimed to critically appraise and synthesise published evidence on the risk factors for poor outcomes in hospitalised COVID-19 patients.

**Methods:**

We searched PubMed, medRxiv and the WHO COVID-19 literature database for studies that reported characteristics of COVID-19 patients who required hospitalisation. We included studies published between January and May 2020 that reported adjusted effect size of any demographic and/or clinical factors for any of the three poor outcomes: mortality, intensive care unit (ICU) admission, and invasive mechanical ventilation. We appraised the quality of the included studies using Joanna Briggs Institute appraisal tools and quantitatively synthesised the evidence through a series of random-effect meta-analyses. To aid data interpretation, we further developed an interpretation framework that indicated strength of the evidence, informed by both quantity and quality of the evidence.

**Results:**

We included a total of 40 studies in our review. Most of the included studies (29/40, 73%) were assessed as “good quality”, with assessment scores of 80 or more. We found that male sex (pooled odds ratio (OR) = 1.32 (95% confidence interval (CI) = 1.18-1.48; 20 studies), older age (OR = 1.05, 95% CI = 1.04-1.07, per one year of age increase; 10 studies), obesity (OR = 1.59, 95% CI = 1.02-2.48; 4 studies), diabetes (OR = 1.25, 95% CI = 1.11-1.40; 11 studies) and chronic kidney diseases (6 studies; OR = 1.57, 95% CI = 1.27-1.93) were associated with increased risks for mortality with the greatest strength of evidence based on our interpretation framework. We did not find increased risk of mortality for several factors including chronic obstructive pulmonary diseases (5 studies), cancer (4 studies), or current smoker (5 studies); however, this does not indicate absence of risk due to limited data on each of these factors.

**Conclusion:**

Male sex, older age, obesity, diabetes and chronic kidney diseases are important risk factors of COVID-19 poor outcomes. Our review provides not only an appraisal and synthesis of evidence on the risk factors of COVID-19 poor outcomes, but also a data interpretation framework that could be adopted by relevant future research.

As of 9 December 2020, the global total number of deaths due to COVID-19 hit 1.5 million. It is imperative to understand the risk factors for COVID-19 mortality as well as other poor outcomes such as intensive care unit (ICU) admission and invasive mechanical ventilation (IMV). This could help identify vulnerable populations who might need prioritisation in terms of protection (eg, “shielding”), prevention (eg, through vaccination), and priority access to hospital-based care. This could also help with the promotion of short-and-longer-term behavioural changes (eg, smoking).

An earlier study [1] identified older age as an important risk factor for COVID-19 mortality, based on 191 patients in Wuhan, China. Some more recent large scale studies [2-4] further identified more risk factors for COVID-19 mortality, such as being male, obesity, diabetes, and cancer. However, these large-scale studies were mainly from the UK and the USA, and there is a need for synthesised evidence incorporating data from all available sources across the globe. To this end, we conducted a systematic review and meta-analysis to synthesise all available evidence regarding the risk factors for poor outcomes in hospitalised COVID-19 patients.

## METHODS

This systematic review is registered with PROSPERO (CRD42020190031) and is reported according to the PRISMA checklist (Appendix S1 in the **Online Supplementary Document**).

### Literature search

The search strategy was designed to identify studies reporting characteristics of patients with COVID-19 who required hospitalisation. We searched PubMed, medRxiv and the WHO COVID-19 literature database (Appendix S2 in the **Online Supplementary Document**) for papers published between January and May 2020. The PubMed search was based on the sensitive COVID-19 string developed by Shokraneh and colleagues [2], combined with a string of MeSH and freetext terms relating to clinical features, study design or outcomes. The search was then adapted for the other databases. The searches were carried out by one reviewer (MD). Only papers in English were considered for inclusion.

### Literature selection

After excluding duplicates, each study was screened by two independent reviewers from a group of six reviewers (TA, AC, ID, MH, EM and AS) using the following selection criteria (**Box 1**). Any disagreements on the selection were resolved through joint discussions among the six reviewers.

Box 1 **Selection criteria**

**Inclusion criteria**

• Reported data for hospitalised COVID-19 infected patients; AND

• Reported data for demographic and/or clinical factors associated with the following poor outcome measures: mortality, ICU admission, and IMV; AND

• Case identification was confirmed by: PCR, nucleic acid test, molecular testing, “laboratory diagnosed/confirmed”; AND

• Reported adjusted OR/RR/HR, with age and sex being considered for adjustment as a minimum; AND

• Used clearly defined demographic and/or clinical factors.

**Exclusion criteria**

• Only included patients who had been hospitalised prior to the outcome(s) of interest; OR

• Definitions were not clear for risk and/or reference group(s); OR

• Reviews, editorials and randomised clinical trials where data in the control arm (placebo group) could not be extracted; OR

• Case series with no comparison group; OR

• Data were reported by another study (in which case the study with the most comprehensive data was included)

### Data extraction

A tailored Excel spreadsheet was used to record data extraction. The following information was collected for each included study: author, publication year, study period and location, study design, outcome(s) of interest, number of patients included, risk group(s) reported and the corresponding reference group, adjusted effect size with confidence interval, etc. Data extraction was conducted by a single reviewer and was checked by a second reviewer from a group of six reviewers (TA, AC, ID, MH, EM and AS). Any disagreements were resolved through joint discussions among the six reviewers, with oversight from YL and HN.

### Quality assessment

For each included study, quality assessment was conducted using the Joanna Briggs Institute Critical Appraisal tool (https://joannabriggs.org/critical-appraisal-tools). Depending on the study design, a questionnaire of ten or eleven questions was completed independently by two reviewers from a group of six reviewers (TA, AC, ID, MH, EM and AS), with any disagreements discussed jointly among the group. Each of the quality assessment questions has four possible answers: yes (indicating good quality), no (indicating bad quality), unknown and not applicable. In order to generate comparable quality scores across different studies, we calculated the percentage of “yes” among all questions (excluding “not applicable”) as the overall score of quality, which could range between 0 and 100. We defined good quality as having a quality assessment score of 80 or more.

### Meta-analysis

#### Main analysis

For each risk factor per outcome (ie, mortality, ICU admission and IMV), we conducted a meta-analysis of odds ratio (OR) only if three or more studies were available. As heterogeneity was expected among studies in terms of study population and methodology, we decided, a priori, to use a random-effects model (using restricted maximum likelihood estimator) for the meta-analysis. Visual inspection of the funnel plot and Egger’s test were used for assessing publication bias.

#### Sensitivity analysis

We considered two sets of sensitivity analyses. Sensitivity analysis 1 excluded studies that had the highest effect size if the pooled effect size in the main analysis was statistically significant (ie, 95% confidence interval did not include 1); this is to exclude the effect of a single outlier that might drive the pooled estimate to the level of statistical significance. Sensitivity analysis 2 excluded studies that had quality scores of less than 80. Similar to the main analysis, sensitivity analyses were conducted only if three or more studies were available.

### Data interpretation criteria

**Figure 1:**
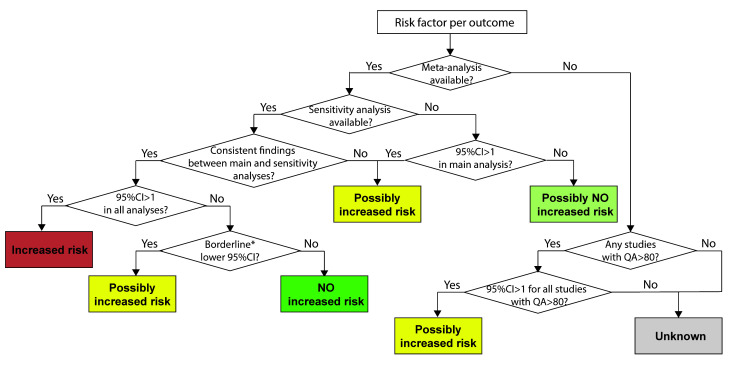
Decision flowchart presenting data interpretation criteria. *Defined as the effect size being between 0.99 and 1.00. CI – confidence interval; QA – quality assessment.

For each risk factor per outcome, a decision framework for data interpretation was designed to assess the strength of the association between these risk factors and outcomes (**Figure 1**). In brief, if a meta-analysis was conducted, the interpretation could be “increased risk”, “possibly increased risk”, “possibly no increased risk” or “no increased risk”, depending on the consistency in the statistical significance of results between the main analysis and sensitivity analyses; the interpretation would be “increased risk” or “no increased risk” only if findings between the main and sensitivity analyses were consistent. When a meta-analysis was not conducted, the interpretation would be either “possibly increased risk” or “unknown”.

### Statistical software

All statistical analyses and visualisation were conducted using R (version 3.6.1) (Foundation for Statistical Computing, Vienna, Austria).

## RESULTS

**Figure 2:**
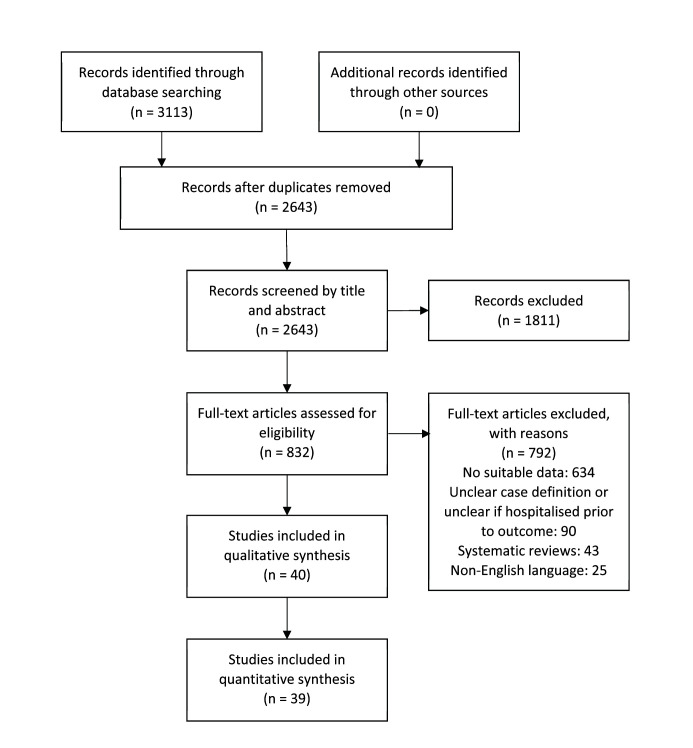
PRISMA flow diagram presenting literature selection process.

After excluding duplicates, 2643 records were screened by title and abstract, leaving 832 records that were further screened by full-text. As a result, a total of 40 studies were included [5-44] in this review (**Figure 2**). We also summarised the prior systematic reviews identified through our literature search (Table S1 in the **Online Supplementary Document**).

Most of the included studies (38/40, 95%) were from China, USA and European countries. As a poor outcome, mortality was reported in 31 studies; ICU admission in eight studies; and IMV in seven studies. Most of the included studies (29/40, 73%) were assessed as “good quality”, with an assessment score of 80 or more (Table S2 in the **Online Supplementary Document**). All studies were included in the meta-analysis with the exception for the study of Mahta et al [27] that focused on angiotensin-converting enzyme inhibitors (ACEi) and angiotensin II receptor blockers (ARB); we did not have enough studies that reported these two factors for meta-analysis. (**Table 1**) No publication bias was indicated in any meta-analyses.

**Table 1:** Characteristics of studies included in the review

**Study**	**Location, Country**	**Study period**	**Mortality**	**ICU**	**IMV**	**Included in meta-analysis**
Alberici, 2020 [6]	Brescia, Italy	Mar 2020	Y	N	N	Y
Al-sabah, 2020 [5]	Kuwait	Feb-May 2020	N	Y	N	Y
Bianchetti, 2020 [7]	Brescia, Italy	Feb-Apr 2020	Y	N	N	Y
Chen, 2020a [9]	Shanghai, China	Jan-Feb 2020	N	Y	N	Y
Chen, 2020b [10]	China	Jan 2020	Y	N	N	Y
Chen, 2020c [8]	Wuhan, China	Jan-Mar 2020	Y	N	N	Y
Chen, 2020d [11]	Wuhan, China	Jan-Mar 2020	Y	N	N	Y
Chroboczek, 2020 [12]	Leman, France	Mar-Apr 2020	N	N	Y	Y
Cummings, 2020 [13]	New York, USA	Mar-Apr 2020	Y	N	N	Y
Docherty, 2020 [14]	UK	Feb-Apr 2020	Y	N	N	Y
Foy, 2020 [15]	USA	Mar-Apr 2020	Y	N	N	Y
Gaibazzi, 2020 [16]	Parma, Italy	Mar 2020	Y	N	N	Y
Gao, 2020 [17]	China	Not reported	Y	N	N	Y
Giacomelli, 2020 [18]	Italy	Feb-Mar 2020	Y	N	N	Y
Giorgi-Rossi, 2020 [19]	Lombardy, Italy	Feb-Apr 2020	Y	N	N	Y
Huang, 2020 [20]	Hubei, China	Jan-Mar 2020	Y	N	N	Y
Hur, 2020 [21]	Chicago, USA	Mar-Apr 2020	N	N	Y	Y
Kalligeros, 2020 [22]	USA	Feb-Apr 2020	N	Y	Y	Y
Kim, 2020 [23]	USA	Mar-May 2020	Y	Y	N	Y
Klang, 2020 [24]	New York, USA	Mar-May 2020	Y	N	N	Y
Li, 2020 [25]	Wuhan, China	Jan-Mar 2020	Y	N	N	Y
Liu, 2020 [26]	China	Feb 2020	Y	N	N	Y
Mehta, 2020 [27]	USA	Mar-Apr 2020	N	Y	Y	N
Murillo-Zamora, 2020 [28]	Mexico	Not reported	Y	N	N	Y
Palaiodimos, 2020 [29]	New York, USA	Mar 2020	Y	N	Y	Y
Petrilli, 2020 [30]	USA	Mar-Apr 2020	Y	N	N	Y
Regina, 2020 [31]	Switzerland	Mar 2020	N	N	Y	Y
Reyes, 2020 [32]	New York, USA	Mar 2020	Y	N	N	Y
Sapey, 2020 [33]	Birmingham, UK	Mar 2020	Y	N	N	Y
Shi, 2020a [36]	Wuhan, China	Jan-Feb 2020	Y	N	N	Y
Shi, 2020b [34]	Wuhan, China	Not reported	Y	Y	N	Y
Shi, 2020c [35]	Wuhan, China	Not reported	Y	Y	N	Y
Simonnet, 2020 [37]	Lille, France	Feb-Apr 2020	N	N	Y	Y
Tang, 2020 [38]	Wuhan, China	Jan-Feb 2020	Y	N	N	Y
Wang, 2020a [39]	Wuhan, China	Jan-Feb 2020	Y	N	N	Y
Wang, 2020b [40]	Changsha, China	Not reported	N	Y	N	Y
Wang, 2020c [41]	Wuhan, China	Not reported	Y	N	N	Y
Xie, 2020 [42]	Wuhan, China	Jan-Feb 2020	Y	N	N	Y
Zhang, 2020a [44]	Wuhan, China	Jan-Feb 2020	Y	N	N	Y
Zhang, 2020b [43]	Wuhan, China; London, UK	Not reported	Y	N	N	Y

ICU – intensive care unit, IMV – invasive mechanical ventilation, Y – yes, N – no

### Risk factors for mortality

#### Risk factors that were associated with increased risk for mortality

**Figure 3:**
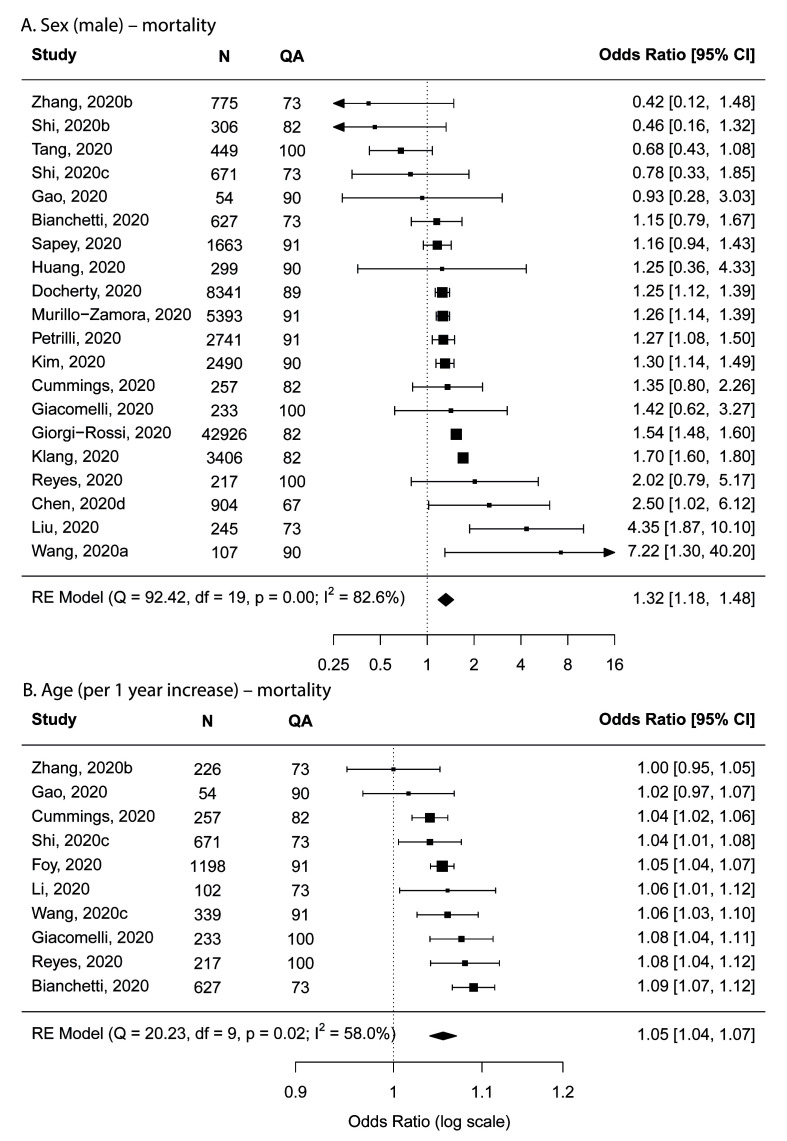
Forest plots showing meta-analysis results for mortality. **Panel A**. Sex. **Panel B**. Age. N – number of subjects, QA – quality assessment score.

Being male was shown to be associated with higher risk for mortality; the pooled OR was 1.32 (95% confidence interval (CI) = 1.18-1.48) in the main analysis based on 20 studies (**Figure 3**), and was 1.31 (95% CI = 1.17-1.47) and 1.32 (95% CI = 1.18-1.47) in two sensitivity analyses.

Two types of studies were available that reported age as a risk factor for mortality: the first type used age as a continuous variable (with no specific reference age group) where the interpretation was the average risk per one year of increase in age among the age range of the study participants; the second type used age groups and selected one age group as the reference. For the first type of study, results from our meta-analysis showed that the pooled OR was 1.05 (95% CI = 1.04-1.07) per one year of age increase in the main analysis based on 10 studies (**Figure 3**), and was 1.05 (95% CI = 1.04-1.06) for both sensitivity analyses. For the second type of study, due to varied reporting of age groups, we were unable to conduct a meta-analysis for any specific age groups but results were broadly consistent with those from the first type of studies (Table S3 in the **Online Supplementary Document**).

**Figure 4:**
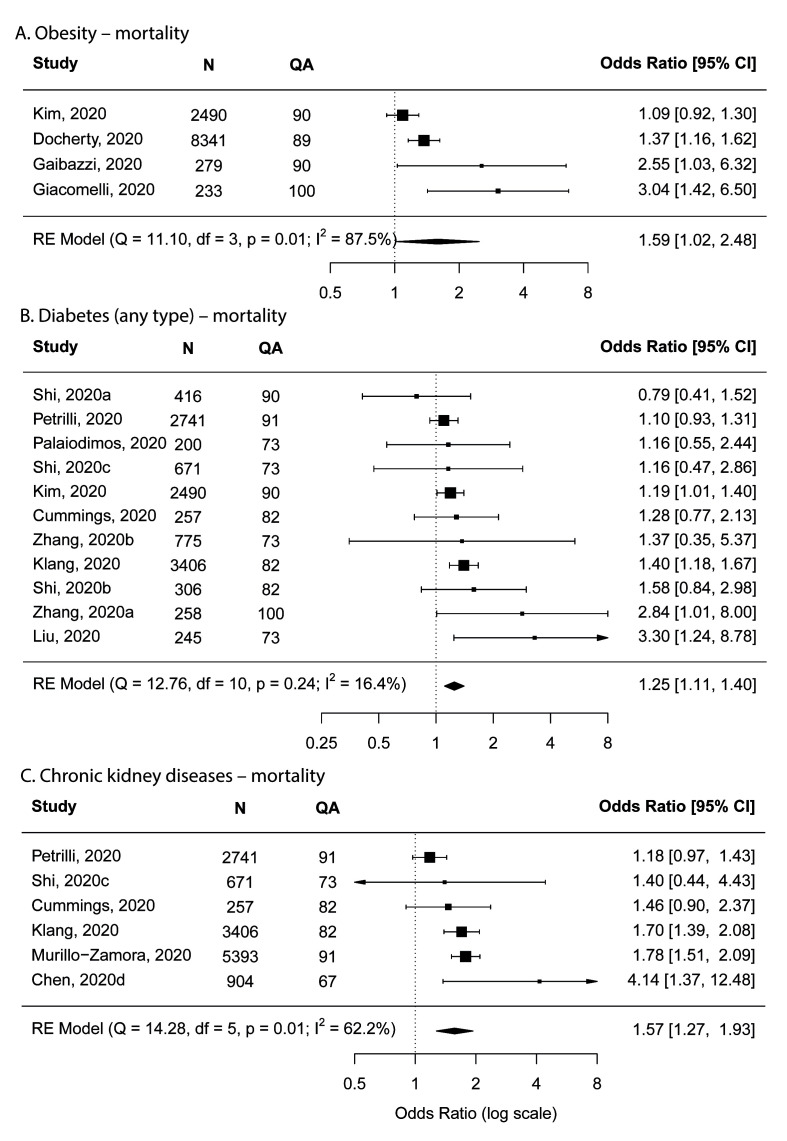
Forest plots showing meta-analysis results for mortality. **Panel A**. Obesity. **Panel B**. Diabetes. **Panel C**. Chronic kidney disease. Obesity is defined as body mass index of >30. N – number of subjects, QA – quality assessment score.

Similar to varied age groupings, BMI was grouped differently among studies and we were only able to conduct a meta-analysis for obesity (BMI>30) compared to non-obesity (BMI<30), based on four studies. Obesity was shown to increase mortality risk with the pooled OR of 1.59 (95% CI = 1.02-2.48) in the main analysis (**Figure 4**) and 1.28 (95% CI = 1.00-1.64) in the sensitivity analysis that excluded the study with the largest effect size. Studies that were not included in the main analysis were summarised in Table S4 in the **Online Supplementary Document**.

Based on the meta-analysis among 11 studies, diabetes (of any type) was shown to increase mortality risk with an OR of 1.25 (95% CI = 1.11-1.40; **Figure 4**). The two sensitivity analyses showed similar results to the main analysis (OR = 1.23, 95% CI: 1.10-1.38 and OR = 1.23, 95% CI = 1.09-1.39). In addition to the 11 studies that reported diabetes of any type and were included in the meta-analysis, the study by Murillo-Zamora et al [28] reported a similar estimate for type-2 diabetes (OR = 1.47, 95% = 1.21-1.56).

Having chronic kidney disease was found to increase mortality risk. Based on six studies, the pooled OR was 1.57 (95% CI = 1.27-1.93) in the main analysis (**Figure 4**), and was 1.52 (95% CI = 1.23-1.88) and 1.57 (95% CI = 1.27-1.93) in the two sensitivity analyses.

#### Risk factors that were possibly associated with increased risk for mortality

**Figure 5:**
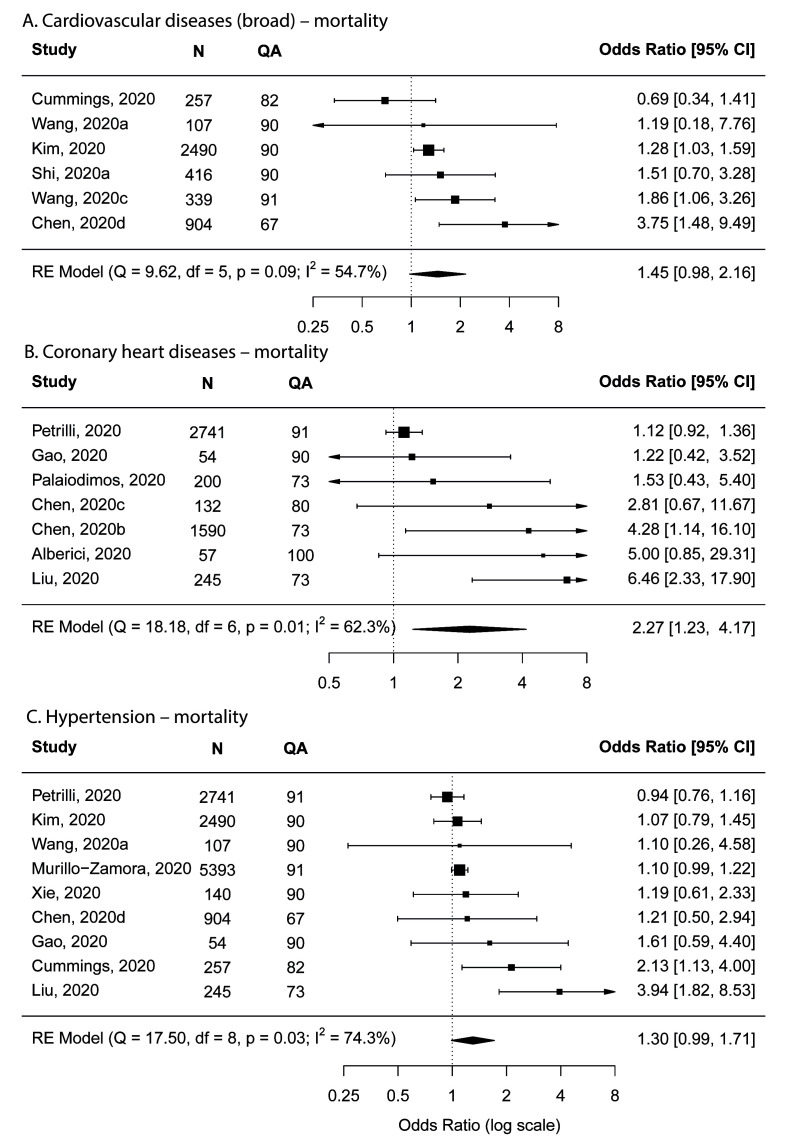
Forest plots showing meta-analysis results for mortality. **Panel A**. Cardiovascular diseases. **Panel B**. Coronary heart diseases. **Panel C**. Hypertension. N – number of subjects, QA – quality assessment score.

Cardiovascular disease and coronary heart disease were both found to be possibly associated with increased risk for mortality; the findings of main and sensitivity analyses were inconsistent. For cardiovascular diseases, the pooled OR in the main analysis was 1.45 (95% CI = 0.98-2.16) based on six studies (**Figure 5**), whereas the pooled OR was 1.29 (95% CI = 1.07-1.55) in the sensitivity analysis that excluded low-quality studies. For coronary heart disease, the pooled OR in the main analysis was 2.27 (95% CI = 1.23-4.17) based on seven studies (**Figure 5**), whereas the pooled OR was 1.40 (95% CI = 0.84-2.32) in the sensitivity analysis that excluded low-quality studies.

Hypertension was also observed to be possibly associated with an increased risk for mortality as borderline lower 95% CI was found in both the main and the sensitivity analysis that excluded low-quality studies, with the pooled OR being 1.30 (95% CI = 0.99-1.71) (**Figure 5**) and 1.09 (95% CI = 0.996-1.19), respectively.

**Figure 6:**
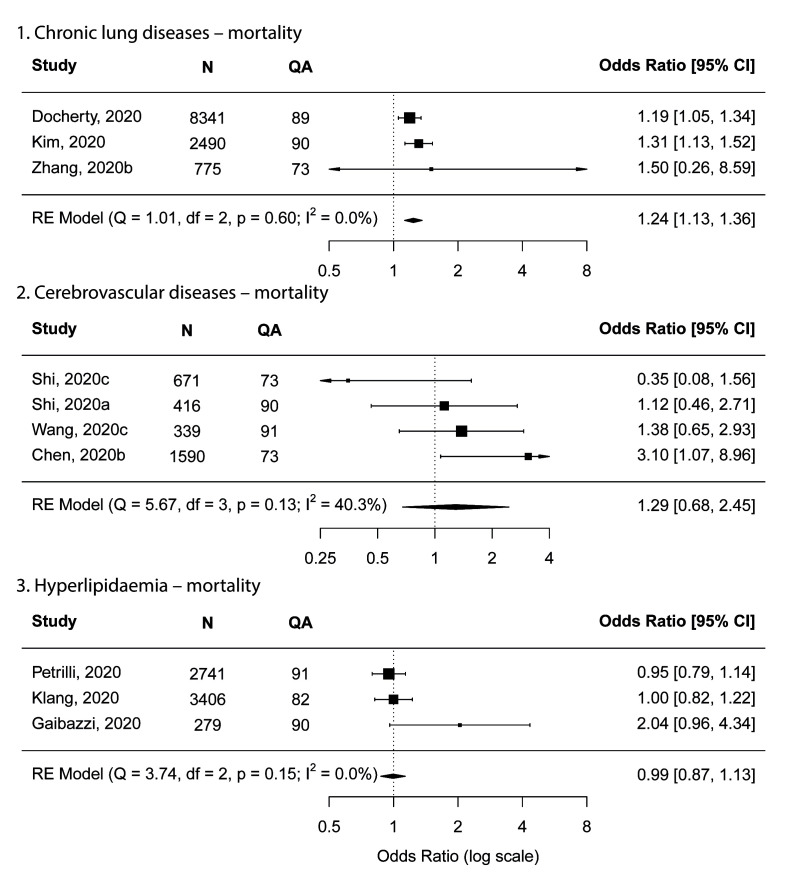
Forest plots showing meta-analysis results for mortality. **Panel A**. Chronic lung diseases. **Panel B**. Cerebrovascular diseases. **Panel C**. Hyperlipidaemia. N – number of subjects, QA – quality assessment score.

Due to limited (only three) availability of studies, only the main meta-analysis was conducted for chronic lung disease, which showed a pooled OR of 1.24 (95% CI = 1.13-1.36), indicating possibly increased risk for mortality (**Figure 6**).

Several other factors were also found to be possibly associated with increased risk for mortality although no meta-analysis could be conducted due to limited studies. These factors include: any comorbidities (as a broad category; one study), heart failure (two studies), any immuno-compromised conditions (as a broad category; one study), malignancy (two studies), any neurological diseases (two studies) and dementia (two studies). Details about these studies are available in Table S5 in the **Online Supplementary Document**.

#### Risk factors that were possibly not associated with increased risk for mortality

Two factors, namely cerebrovascular diseases and hyperlipidaemia, were possibly not associated with increased risk for mortality with 95% CI including one, based on four studies and three studies, respectively (**Figure 6**). No sensitivity analyses could be done due to limited studies.

#### Risk factors that were not associated with increased risk for mortality

**Figure 7:**
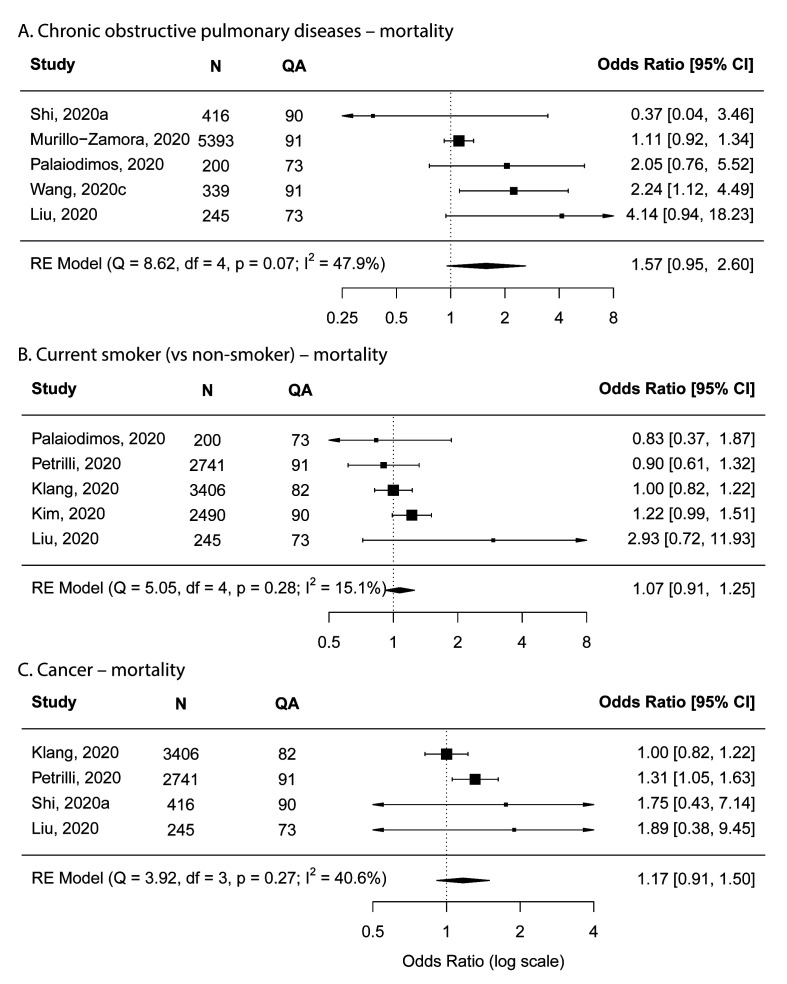
Forest plots showing meta-analysis results. **Panel A**. Chronic obstructive pulmonary diseases. Panel B. Current smoker. **Panel C**. Cancer. N – number of subjects; QA – quality assessment score.

Consistent meta-analysis results were observed between the main analysis and sensitivity analyses for chronic obstructive pulmonary diseases (COPD), cancer and current smoker, which indicated that these factors were not associated with increased risk for mortality (**Figure 7**).

### Risk factors for ICU admission

Being male was associated with an increased risk for ICU admission and the corresponding pooled OR was 1.82 (95% CI = 1.09-3.03) based on five studies in the main analysis (**Figure S1** in the Online Supplementary Document), and was 1.35 (95% CI = 1.21-1.51) and 1.34 (95% CI = 1.20-1.50) in the two sensitivity analyses. Several factors were possibly associated with increased risk for ICU admission (**Table S6** in the **Online Supplementary Document**): older age (two studies), obesity (one study), use of ACEi (one study) and any immuno-compromised condition (one study).

Diabetes was possibly not associated with increased risk for ICU admission although only the main analysis was conducted among three studies (OR = 2.24, 95% CI = 0.84-5.95). Hypertension was not associated with increased risk for ICU admission; the pooled OR was 0.90 (95% CI = 0.78-1.04) based on six studies in the main analysis and was 0.91 (95% CI = 0.79-1.06) in the sensitivity analysis that excluded low-quality studies (Figure S1 in the **Online Supplementary Document**).

### Risk factors for IMV

Being male and diabetes were associated with increased risk for IMV; the pooled OR was 2.20 (95% CI = 1.50-3.23) based on six studies and was 1.59 (95% CI = 1.10-2.29) based on four studies, respectively (Figure S2 in the **Online Supplementary Document**). Older age and BMI>35 were possibly associated with increased risk for IMV, based on one and three studies, respectively (Table S7 in the **Online Supplementary Document**).

Hypertension was possibly not associated with increased risk for IMV; the pooled OR was 1.45 (95% CI = 0.42-4.98) based on three studies (Figure S2 in the **Online Supplementary Document**).

## DISCUSSION

To our knowledge, this is one of the most comprehensive systematic reviews that assessed the full spectrum of risk factors for poor outcomes among hospitalised COVID-19 patients. We critically appraised and synthesised all best available evidence from both peer-reviewed publications and non-peer-reviewed preprints. We also developed a data interpretation framework that accounted for the strength of evidence and ensured consistency and comparability across risk factors and poor outcomes. Based on the framework, we found that being male, older age, obesity, diabetes and chronic kidney diseases were associated with risks for poor outcomes with the greatest strength of evidence. The full list of reported risk factors with different levels of evidence strength is available in **Table 2**.

**Table 2:** Summary of data synthesis interpretations of risk factors for poor outcomes among hospitalised COVID-19 patients

Poor outcome	Increased risk (with pooled OR)	Possibly increased risk	Possibly no increased risk*	No increased risk*
Mortality	• Male (20 studies; OR = 1.32, 95% CI = 1.18-1.48)	• Any comorbidities (1 study)	• Cerebrovascular disease (4 studies)	• COPD (5 studies)
	• Older age (10 studies; OR = 1.05 95% CI = 1.04-1.07, per one year of age increase)	• Any cardiovascular diseases (6 studies)	• Hyperlipidaemia (3 studies)	• Cancer (4 studies)
	• Obesity (4 studies; OR = 1.59, 95% CI = 1.02-2.48)	• Coronary heart disease (7 studies)		• Current smoker (5 studies)
	• Diabetes (11 studies; OR = 1.25, 95% CI = 1.11-1.40)	• Hypertension (9 studies)		
	• Chronic kidney disease (6 studies; OR = 1.57,95% CI = 1.27-1.93)	• Heart failure (2 studies)		
		• Chronic lung disease (3 studies)		
		• Any immuno-compromised condition (1 study)		
		• Malignancy (2 studies)		
		• Any neurological diseases (2 studies)		
		• Dementia (2 studies)		
ICU admission	• Male (5 studies; OR = 1.82, 95% CI = 1.09-3.03)	• Older age (2 studies)	• Diabetes (3 studies)	• Hypertension (6 studies)
		• Obesity (1 study)		
		• Use of ACEi (1 study)		
		• Any immuno-compromised condition (1 study)		
Invasive mechanical ventilation	• Male (6 studies; OR = 2.20, 95% CI = 1.50-3.23)	• Older age (1 study)	• Hypertension (3 studies)	
	• Diabetes (4 studies; OR = 1.59, 95% CI = 1.10-2.29)	• BMI>35 (2 studies)		

OR – odds ratio, BMI – body mass index, COPD – chronic obstructive pulmonary disease, ACEi – angiotensin-converting-enzyme inhibitor

*Interpret with caution: risk factors that apparently show no association with outcome could be due to lack of statistical power.

Although several factors, such as COPD, cancer, current smoker and hypertension, were interpreted as not being associated with increased risk for poor outcomes, it should be noted that this is based on currently available evidence that might lack the necessary statistical power. As a result, we caution against any interpretations that these characteristics are not risk factors. For those findings that were interpreted as being (definitively) or possibly being associated with increased risk for poor outcomes, we caution against interpreting these effects as causal effects.

We reported that there was an increased risk of 5% (95% CI = 4-7) for mortality given one year of age increase, which was based on studies that included age as a continuous variable in their models. Although this indicated that older age was an important risk factor, the finding did not necessarily imply that there was a linear relationship between age and risk for mortality. With this caveat, we caution against any quantitative interpretations of our findings (eg, 5% of increase in the example above) regarding age.

Our study has several strengths. First, we limited our selection criteria to COVID-19 patients that were laboratory-confirmed to reduce misclassification bias at study level. Second, we limited our selection criteria to studies that reported the adjusted effect (eg, through multivariate analysis) to reduce confounding at study level. Third, we selected not to include composite poor outcomes that often varied by study to ensure comparability. Fourth, to address potential publication bias, we included evidence from both peer-reviewed and non-peer-reviewed sources and appraised the quality of all evidence. Finally, we developed a data interpretation framework that was informed by both quantity and quality of the evidence.

Our study does have limitations. First, we focused on hospitalised COVID-19 patients. This could lead to biased estimates of risk for some risk factors, in particular when access to health care differed between those with that risk factor and those without. For example, patients with certain comorbidities might be more likely to be hospitalised with COVID-19 symptoms than those without. This means that the clinical severity of COVID-19 among hospitalised patients with certain comorbidities could be overall lower than those without certain comorbidities, resulting in selection bias towards null hypothesis (if these comorbidities truly increase risk for poor outcomes). Second, as an evidence review from a global perspective, we were unable to account for local contexts in our analysis, such as social distancing and lockdowns, access to health care (especially at the beginning of the pandemic) and access to personal protective equipment. These local contexts could vary greatly across regions and might contribute to the heterogeneity of the findings. Third, we were unable to assess effect-modifiers due to lack of relevant data. For example, age and/or sex could be effect modifier(s) for some comorbidities [45]. Fourth, we did not include non-English papers in our review. Fifth, our searches yielded prior systematic reviews but we did not have the resource to scrutinise the included studies for inclusion in this review; we have summarised those prior reviews in the appendix (Table S1 in the **Online Supplementary Document**). Finally, we only included data published between January and May 2020, and it is likely that inclusion of data from studies published later could alter some of our findings and interpretation.

## CONCLUSIONS

In conclusion, our review provides not only an appraisal and synthesis of evidence on the risk factors of COVID-19 poor outcomes, but also a data interpretation framework that could be adopted by relevant future research.

## Additional material

Online Supplementary Document
