# Supplementary material for: Risk factors for poor outcomes in hospitalised COVID-19 patients: A systematic review and meta-analysis
Source: J Glob Health. 2021 Mar 1;11:10001. doi: 10.7189/jogh.11.10001 (PMC7980087; doi:10.7189/jogh.11.10001)
Supplement: Online Supplementary Document [file jogh-11-10001-s001.pdf]

Contents

Appendix S1. PRISMA Checklist 2

Appendix S2. Search strategy 4

Table S1. Summary of relevant systematic literature reviews identified by the literature search 6

Table S2. Quality assessment results using Joanna Briggs Institute Critical Appraisal Tools 16

Table S3. Summary of findings from studies not included in the meta-analysis: age and mortality 18

Table S4. Summary of findings from studies not included in the meta-analysis: BMI and mortality 19

Table S5. Summary of findings from studies not included in the meta-analysis: other risk factors and mortality 20

Table S6. Summary of findings from studies not included in the meta-analysis: risk factors and ICU admission 21

Table S7. Summary of findings from studies not included in the meta-analysis: risk factors and invasive mechanical ventilation 22

Figure S1. Forest plots showing meta-analysis results of risk factors for ICU admission 23

Figure S2. Forest plots showing meta-analysis results of risk factors for invasive mechanical ventilation 24

Reference 25

Appendix S1. PRISMA Checklist

Section/topic	#	Checklist item	Reported on page #
**TITLE**			
Title	1	Identify the report as a systematic review, meta-analysis, or both.	1
**ABSTRACT**			
Structured summary	2	Provide a structured summary including, as applicable: background; objectives; data sources; study eligibility criteria, participants, and interventions; study appraisal and synthesis methods; results; limitations; conclusions and implications of key findings; systematic review registration number.	1
**INTRODUCTION**			
Rationale	3	Describe the rationale for the review in the context of what is already known.	2
Objectives	4	Provide an explicit statement of questions being addressed with reference to participants, interventions, comparisons, outcomes, and study design (PICOS).	2
**METHODS**			
Protocol and registration	5	Indicate if a review protocol exists, if and where it can be accessed (e.g., Web address), and, if available, provide registration information including registration number.	2
Eligibility criteria	6	Specify study characteristics (e.g., PICOS, length of follow-up) and report characteristics (e.g., years considered, language, publication status) used as criteria for eligibility, giving rationale.	2-3
Information sources	7	Describe all information sources (e.g., databases with dates of coverage, contact with study authors to identify additional studies) in the search and date last searched.	2; appendix
Search	8	Present full electronic search strategy for at least one database, including any limits used, such that it could be repeated.	2; Appendix
Study selection	9	State the process for selecting studies (i.e., screening, eligibility, included in systematic review, and, if applicable, included in the meta-analysis).	3
Data collection process	10	Describe method of data extraction from reports (e.g., piloted forms, independently, in duplicate) and any processes for obtaining and confirming data from investigators.	3
Data items	11	List and define all variables for which data were sought (e.g., PICOS, funding sources) and any assumptions and simplifications made.	3
Risk of bias in individual studies	12	Describe methods used for assessing risk of bias of individual studies (including specification of whether this was done at the study or outcome level), and how this information is to be used in any data synthesis.	3
Summary measures	13	State the principal summary measures (e.g., risk ratio, difference in means).	3
Synthesis of results	14	Describe the methods of handling data and combining results of studies, if done, including measures of consistency (e.g., I^2) for each meta-analysis.	3

Section/topic	#	Checklist item	Reported on page #
Risk of bias across studies	15	Specify any assessment of risk of bias that may affect the cumulative evidence (e.g., publication bias, selective reporting within studies).	3
Additional analyses	16	Describe methods of additional analyses (e.g., sensitivity or subgroup analyses, meta-regression), if done, indicating which were pre-specified.	3
**RESULTS**			
Study selection	17	Give numbers of studies screened, assessed for eligibility, and included in the review, with reasons for exclusions at each stage, ideally with a flow diagram.	Figure 2
Study characteristics	18	For each study, present characteristics for which data were extracted (e.g., study size, PICOS, follow-up period) and provide the citations.	4; Table 1
Risk of bias within studies	19	Present data on risk of bias of each study and, if available, any outcome level assessment (see item 12).	4; Table S2
Results of individual studies	20	For all outcomes considered (benefits or harms), present, for each study: (a) simple summary data for each intervention group (b) effect estimates and confidence intervals, ideally with a forest plot.	Figures 3–7 and Figures S1–2
Synthesis of results	21	Present results of each meta-analysis done, including confidence intervals and measures of consistency.	Figures 3–7 and Figures S1–2
Risk of bias across studies	22	Present results of any assessment of risk of bias across studies (see Item 15).	4
Additional analysis	23	Give results of additional analyses, if done (e.g., sensitivity or subgroup analyses, meta-regression [see Item 16]).	4–6
**DISCUSSION**			
Summary of evidence	24	Summarize the main findings including the strength of evidence for each main outcome; consider their relevance to key groups (e.g., healthcare providers, users, and policy makers).	6
Limitations	25	Discuss limitations at study and outcome level (e.g., risk of bias), and at review-level (e.g., incomplete retrieval of identified research, reporting bias).	7
Conclusions	26	Provide a general interpretation of the results in the context of other evidence, and implications for future research.	7
**FUNDING**			
Funding	27	Describe sources of funding for the systematic review and other support (e.g., supply of data); role of funders for the systematic review.	7

Appendix S2. Search strategy

Pubmed [1]

Searched 2020-05-26

1842 results

((((patient*[Title/Abstract] AND data[Title/Abstract]) OR "Clinical Features" OR review OR cohort OR "case series" OR "case report*" OR observational OR "cross sectional" OR “retrospective study” OR "risk factors")) AND (((mortality OR hospitalis* OR hospitaliz* OR intubation OR ventilation OR admission* OR admitted OR "critical care" OR "critical cases" OR death OR "severe cases")[Title/Abstract]) OR mortality[MeSH Subheading])) AND (("Betacoronavirus"[Mesh] OR "Coronavirus Infections"[MH] OR "Spike Glycoprotein, COVID-19 Virus"[NM] OR "COVID-19"[NM] OR "Coronavirus"[MH] OR "Severe Acute Respiratory Syndrome Coronavirus 2"[NM] OR 2019nCoV[ALL] OR Betacoronavirus*[ALL] OR Corona Virus*[ALL] OR Coronavirus*[ALL] OR Coronovirus*[ALL] OR CoV[ALL] OR CoV2[ALL] OR COVID[ALL] OR COVID19[ALL] OR COVID-19[ALL] OR HCoV-19[ALL] OR nCoV[ALL] OR "SARS CoV 2"[ALL] OR SARS2[ALL] OR SARSCoV[ALL] OR SARS-CoV[ALL] OR SARS-CoV-2[ALL] OR Severe Acute Respiratory Syndrome CoV*[ALL]) AND ((2019/11/17[EDAT] : 3000[EDAT]) OR (2019/11/17[PDAT] : 3000[PDAT])))

medRxiv via https://mcguinlu.shinyapps.io/medrxivr/

Date limits: record creation date 20200101 to present

Searched 2020-05-26

605 results

Topic clusters below combined internally with OR and between clusters with AND

Covid cluster

COVID-19

[Cc]oronavirus

SARS-CoV-2

2019-nCoV

Population cluster

[Mm]ortality

[Dd]eath

[Hh]ospital

[Ii]ntubat

[Vv]entilation

[Aa]dmission

[Aa]dmitted

Publication / study type cluster

[Rr]eview

[Cc]ohort

[Cc]ase series

[Cc]ase report

[Oo]bservational

[Cc]ross sectional

[Rr]etrospective

[Cc]linical characteristics

[Rr]isk factor

WHO COVID-19 literature database

https://search.bvsalud.org/global-literature-on-novel-coronavirus-2019-ncov/?lang=en

Searched 2020-05-26

615 results

(tw:(mortality OR hospitalis* OR hospitaliz* OR intubation OR ventilation OR admission* OR admitted)) AND (tw:(review OR cohort OR "case series" OR "case report*" OR observational OR "cross sectional" OR "cross-sectional"))

Table S1. Summary of relevant systematic literature reviews identified by the literature search

Study	Objective(s)	Main finding(s)	Limitation(s)
**Agganwal et al. 2020 [2]**	• To provide a more robust evidence base in respect of the links between cardiovascular disease and COVID-19 outcomes.	• Pre-existing CVD was significantly associated with increased risk of severe COVID-19, and increased risk of COVID-19 all-cause mortality (but not specifically with increased risk of mortality among subjects with severe disease).	• Majority of studies were from China (16, +2 from USA).<br>• Studies up to 20 April 2020 were included.<br>• Composite outcome measure ('severity') used.
**Aghagoli et al. 2020 [3]**	• To gather and distil the existing body of literature that describes the cardiac implications of COVID-19.	• COVID-19 patients with pre-existing cardiovascular disease were more in intensive care unit settings, with greater rates of mortality.<br>• Studies have noted cardiac presentations for COVID-19, rather than respiratory, such as acute pericarditis and left ventricular dysfunction. In some patients there has been evidence of acute myocardial injury, with correspondingly increased serum troponin I levels.	• Lack of data on cardiac surgical interventions, particularly data describing myocardial protection during cardiac surgery for COVID-19 patients.<br>• This review also had very little description of methods and nothing on quality assessment.<br>• No meta-analysis
**Alqahtani et al. 2020 [4]**	• Severity and mortality risks of COVID-19 in patients with COPD and smokers	• Pooled prevalence of COPD was 2%.<br>• RR of mortality using two studies on mortality was 1.10 (CI 0.6-1.8).<br>• Pooled prevalence of smokers was 9%.<br>• RR of mortality using 2 studies was 1.45 (CI 1.03-2.04).	• Studies up to 24 March 2020.<br>• All conducted in China apart from one from USA.<br>• Severity of COPD only stated in 7 studies. Pooled prevalence calculated but mortality only given in 2 studies.
**Baral et al. 2020 [5]**	• To assess the association between ACEi and ARB with critical event and mortality in COVID-19	• 18.3% patients were prescribed ACEi/ARB but were not associated with critical events or mortality	• Wide definitions of critical and severe in studies, varied population admitted to hospital.

Study	Objective(s)	Main finding(s)	Limitation(s)
**Bellou et al. 2020 [6]**	• To identify clinical risk factors associated with poor COVID-19 outcomes	• Risk factors associated with severer COVID-19 include elevated C-reactive protein, decreased lymphocyte count, cerebrovascular disease, COPD, diabetes mellitus, haemoptysis & male sex.	• Studies up to 19 April 2020; limited to PubMed.<br>• Authors reported that most ORs are unadjusted, so confounding not ruled out.<br>• Most studies from China.
**Bezabih et al. 2020 [7]**	• To assess the effects of RAAS inhibitors on COVID-19 outcomes	• Risk of poor COVID-19 outcomes was lower among those taking RAAS inhibitors, compared to those taking non-RAAS-inhibiting anti-hypertensive.	• Only 7 studies eligible for inclusion.<br>• Authors reported limited adjustment for confounding in the studies, and possible publication bias.<br>• Authors also noted that choice of anti-hypertensive medication tends to depend on presence of other comorbidities.
**Bin Abdulhak et al. 2020 [8]**	• Effects of prior use of ACEi/ARB on outcome of COVID-19 patients	• Prior use of ACEi/ARB associated with reduction of inpatient mortality from pooled adjusted OR from 4 studies and reduction of critical or fatal outcome	• Evidence from limited number of studies
**Gao et al. 2020 [9]**	• Prevalence of cancer in patients with COVID-19 and whether there is an association with severe illness and mortality	• Cancer was associated with severe illness for and mortality in patients with COVID-19. Included studies from Italy, France, Korea and China.	• Authors did not evaluate type of cancer.
**Ghosal et al. 2020 [10]**	• Effect of ACEi/ARB on severity of COVID-19, risk of hospitalisation and death compared to those not on ACEi/ARB	• Safe to use ACEi/ARB and reduce risk of death from 4 papers. Severity and risk of hospitalisation not statistically significant.	• Analysis limited by confounding factors, various endpoint measured by the studies, only four studies used for mortality OR.

Study	Objective(s)	Main finding(s)	Limitation(s)
**Grover et al. 2020 [11]**	• Assess the outcomes of patients with COVID-19 who are taking ACEi/ARB	• Pooled analysis of 9 studies showed reduction risk of death for those on ACEi/ARB compared to those not on these medications	• Diversity of studies included. Only pooled meta-analysis of 9 out of the 16 studies for mortality and 5 for severity with non-uniformity regarding definition of severity<br>• No quality assessment.
**Hage et al. 2020 [12]**	• To evaluate the links between immunosuppression among solid organ transplant (SOT) recipients, COVID-19 infection, and COVID-19 outcomes.	• Given limited evidence base, no firm conclusions could be drawn.<br>• Suggested that immunosuppression among SOT recipients might lead to better COVID-19 outcomes, because it prevents hyper-inflammation (cytokine storm).	• Only 5 studies eligible for inclusion.
**Hessami et al. 2020 [13]**	• To analyse the burden of cardiovascular disease among patients with COVID-19, focusing on studies which reported CVD among hospitalised patients with COVID-19, with mortality & ICU admission as primary outcomes.	• Acute cardiac injury, hypertension, heart failure, other CVD, and overall CVD are all significantly associated with mortality in COVID-19.<br>• Arrhythmia, acute cardiac injury, coronary heart disease, CVD and hypertension are also significantly associated admission to ICU.<br>• Risks for ICU admission significantly higher in males than in females.	• Authors reported high heterogeneity of primary studies as a limitation, and noted that they had not been able to consider confounding effects of other (non-CVD) conditions on outcomes.<br>• Most studies in meta-analysis were carried out in China only.
**Hu et al. 2020 [14]**	• To evaluate the risk factors of COVID-19.	• The most prevalent comorbidities were hypertension and diabetes, which were associated with the severity of COVID-19.	• Studies up to 10 March 2020, all from China and 1 from Singapore.

Study	Objective(s)	Main finding(s)	Limitation(s)
**Huang et al. 2020a [15]**	• To investigate the association between diabetes and composite poor outcome (incl. mortality, severe illness, ARDS, ICU admission & disease progression)	• Diabetes was associated with composite poor outcome, mortality, severe illness and disease progression but not ICU admission. It was affected by age, HTN but not sex, CVD and COPD.	• Composite outcome<br>• Early studies mostly in China (until 8th April)<br>• Did not specify PCR diagnosed or hospitalised patients<br>• Authors noted that the subjects might overlap
**Huang et al. 2020b [16]**	• To investigate the link between lymphocyte count on admission and severity of COVID-19 outcomes.	• Patients with poor COVID-19 outcomes had a lower lymphocyte count than those with good outcomes.	• Authors reported likely presence of publication bias.<br>• Most studies included were pre-prints, and most exclusively from China - possibility of overlapping patient groups.
**Islam et al. 2020 [17]**	• To investigate the link between comorbidities and other clinical characteristics, and death from COVID-19	• Male sex, age over 50, and presence of comorbidities are all associated with higher risk of death from COVID-19.	• Most studies were from China<br>• Follow-up of patients varied from study to study<br>• High heterogeneity among studies.
**Jain et al. 2020 [18]**	• To identify symptoms and comorbidities predictive of COVID-19 severity	• More males admitted to ICU, dyspnoea only significant symptoms predictive of severe disease and ICU admission. COPD, CVD, HTN were predictive of severity & ICU	• No consistent definition of severe, time at which severity was determined unclear, not clear how symptoms or co-morbidity was measured, only univariate analysis carried out due to lack of quality of data and missing data

Study	Objective(s)	Main finding(s)	Limitation(s)
**Jutzeler et al. 2020 [19]**	• To gather all available information about comorbidities, clinical signs & symptoms, outcomes, lab findings, imaging features and treatments in COVID-19 patients.	• Older age, being male, and pre-existing comorbidity are risk factors of in-hospital mortality.	• Studies to 28 March 2020<br>• Not clear if all subjects had lab-confirmed COVID-19<br>• Not clear if all subjects were hospitalised.<br>• Significant heterogeneity between studies. Some risk of publication bias reported.
**Kumar et al. 2020 [20]**	• To assess the influence of diabetes on COVID-19 severity and mortality.	• COVID-19 subjects with diabetes have 2 fold greater risk of severe disease or death than those without diabetes.	• Authors reported it was not possible to establish whether there is an independent link between diabetes and COVID-19 outcomes or whether it is a confounding factor (linked to age, weight, comorbidities); or whether there is a particular link with poor glycaemic control and poor COVID-19 outcomes.
**Li et al. 2020 [21]**	• To determine the association between cardiovascular metabolic diseases and the development of COVID-19.	• At least 8% of patients with COVID-19 had acute cardiac injury.<br>• Incidence of acute cardiac injury was 13 fold higher in patients admitted to ICU / with severe COVID-19.	• Only 6 studies eligible for inclusion.<br>• Unclear whether all subjects were hospital-based, or whether all subjects had lab-confirmed COVID-19.
**Liguoro et al. 2020 [22]**	• To study clinical characteristics of COVID-19 among children aged under 18	• Children were less likely to develop severe symptoms of COVID-19 than adults.	• Authors reported that larger studies were needed to explore effects of COVID-19 among children.

Study	Objective(s)	Main finding(s)	Limitation(s)
**Lippi et al. 2020 [23]**	• To evaluate the link between hypertension and severe or fatal COVID-19	• Hypertension was associated with a 2.5 fold greater risk of COVID-19 as well as a significantly higher risk of mortality.	• Studies up to 26 March 2020<br>• No quality assessment carried out
**Liu et al. 2020 [24]**	• To understand the association between ACEi and ARBs, and COVID-19.	• No significant increase in COVID-19 infection among patients with ACEI or ARB therapy; decreased risk of severe COVID-19 or death among patients with ACEI or ARB therapy.	• Unclear if all patients had lab-confirmed COVID-19, or were hospitalised.
**Ludvigsson et al. 2020 [25]**	• To establish data on symptoms and prognosis of COVID-19 among children.	• Children account for 1-5% of COVID-19 cases, often have milder disease than adults, and deaths among children are extremely rare.	• Studies up to 18 March 2020; included all COVID-19 cases (not only hospitalised cases), with majority of studies from China<br>• No second reviewer.
**Martins-Filho et al. 2020 [26]**	• To inform on the management of critically ill COVID-19 patients by performing a meta-analysis of clinical and lab factors associated with mortality in COVID-19 patients.	• Older age, presence of chronic inflammatory states, dyspnea at disease onset, among other factors were important predictors for mortality in patients with COVID-19.<br>• Also an increased risk of death for patients who develop ARDS, cardiac injury, acute kidney disease, DIC, and sepsis.	• High number of studies with potential overlapping data which compromise the strength of available evidence<br>• Univariate analysis only.
**Mason et al. 2020 [27]**	• To understand the age adjusted relationship between co-morbidities and COVID-19 outcome	• Obesity, HTN, DM, COPD and cancer were associated with worse outcomes and those with multiple co-morbidities had more than twice the risk of severe outcome or death	• Narrative synthesis<br>• Methods for data collection not well reported<br>• Variation in definitions and outcomes
**Matsushita et al. 2020 [28]**	• To assess the relationship of cardiovascular risk factors with severity of COVID-19	• Hypertension, diabetes, CVD, male and age associated with severe COVID-19.	• Only four studies adjusted for age and sex, mostly Chinese studies and possibility that some patients included in multiple studies

Study	Objective(s)	Main finding(s)	Limitation(s)
**Nasiri et al. 2020 [29]**	• To identify baseline characteristics of COVID-19 patients	• Males had 3 fold higher risk of mortality compared with females	• Studies up to 3 March 2020<br>• All studies except one (Germany) based in China<br>• No discussion of the quality of the papers.
**Parohan et al. 2020a [30]**	• To identify risk factors of mortality from COVID-19	• Older age, hypertension, diabetes, COPD and CVDs were associated with risk of death based on HR, OR or RR	• Only 6 studies included
**Parohan et al. 2020b [31]**	• To summarize available findings on the association between liver injury and severity of COVID-19 infection	• Higher serum levels AST, ALT, total bilirubin and albumin levels found to be associated with a significant increase in the severity of COVID-19 infection	• Excluded data on hepatitis infections and limited by small sample size.
**Pigoga et al. 2020 [32]**	• To identify predictors of severe COVID-19	• Age, lymphocyte count, hypertension and history of any pre-existing medical condition among other factors were associated with severe disease	• Numerous proxy measures of illness severity<br>• No meta-analysis.
**Pranata et al. 2020a [33]**	• To evaluate the association between cerebrovascular, and cardiovascular diseases and poor outcome in patients with COVID-19 pneumonia using a qualitative synthesis and meta-analysis.	• Cerebrovascular disease was associated with an increased composite poor outcome.<br>• Subgroup analysis revealed that cerebrovascular disease was associated with mortality and showed borderline significance for severe COVID-19.<br>• Cardiovascular disease was associated with increased composite poor outcome, mortality, and severe COVID-19.<br>• Association was not influenced by gender, age, hypertension, diabetes, and respiratory comorbidities.	• High number of pre-prints and retrospective in design, also a high risk for publication bias.<br>• Majority of studies from China. Information regarding the use of chronic medications such as ACEI/ARB is lacking.

Study	Objective(s)	Main finding(s)	Limitation(s)
**Pranata et al. 2020b [34]**	• To assess the association between N-terminal pro-brain natriuretic peptide (NT-proBNP) and mortality in patients with COVID-19.	• NT-proBNP was higher in non-survivor group. Elevated NT-proBNP was associated with increased mortality.	• Risk of publication bias in included studies, high number of pre-prints, small sample size, and varied cut-off points for NT-proBNP levels.
**Pranata et al. 2020c [35]**	• To investigate the association between hypertension and outcome in patients with COVID-19 pneumonia.	• Hypertension associated with severity, ARDS, ICU admission, and death.<br>• Meta-regression analysis showed that gender was a covariate that affects the association.	• Data on ACEi/ARB use not included, most studies were retrospective, originated from China (potential for data overlap), and were pre-prints.
**Roncon et al. 2020 [36]**	• To assess the risk of ICU admission and mortality risk in diabetic COVID-19 patients	• Diabetes resulted to be the second more frequent comorbidities.<br>• Diabetic patients resulted to have a significant increased risk of ICU admission.<br>• In 471 patients analysed for the secondary outcome diabetic subjects resulted to be at higher mortality risk.	• Only 8 studies in total, 4 for the primary outcome (ICU admission) and 4 for secondary outcome (death).
**Ssentongo et al. 2020 [37]**	explore the association of pre-existing nditions with COVID-19 mortality	ronary heart disease, hypertension, congestive art failure, and cancer significantly increased the k of mortality from COVID-19.	able to explore the influence that ncer, HIV, and asthma might have on VID-19 mortality.
**Tamara et al. 2020 [38]**	• To understand whether obesity was related to poor outcome for CV 19 patients	• Three studies reported OR for developing severe disease. The OR reported by the paper with the highest quality for mechanical ventilation of 7.36 (CI 1.63 - 33.14) for those with BMI > 35.	• Studies up to 14 April 2020.<br>• Only three studies included though all high quality.<br>• Different BMI definitions for obesity.

Study	Objective(s)	Main finding(s)	Limitation(s)
**Tian et al. 2020 [39]**	• To evaluate the risk factors associated with mortality in COVID-19	• The presence of comorbidities such as hypertension, coronary heart disease, and diabetes were associated with significantly higher risk of death amongst COVID-19 patients.<br>• Those who died, compared to those who survived, differed on multiple biomarker levels on admission including elevated levels of cardiac troponin; C-reactive protein; interleukin6; D-dimer; creatinine and alanine transaminase; as well as decreased levels of albumin.	• All but one study were from China, limiting generalizability across populations.
**Vardavas et al. 2020 [40]**	• To evaluate the association between smoking and COVID-19 outcomes including the severity of the disease, the need for mechanical ventilation, and the need for intensive care unit hospitalization and death.	• Smoking is most likely associated with the negative progression and adverse outcomes of COVID-19.	• All studies conducted in China, no-meta analysis.
**Zhao et al. 2020 [41]**	• To analyse the risk factors associated with COVID-19 severity.	• Predictors for disease severity include age (over 50 years), male, smoking, and any comorbidity (e.g. CKD, COPD, cerebrovascular disease), as well as increased LHD, CRP or D-dimer, and decreased blood platelet or lymphocyte counts. Old age, cardiovascular disease, hypertension & diabetes were independent predictors of COVID-19 death.	• Studies up to 25 February 2020. Majority of the studies were in Wuhan or other cities in China.<br>• Authors noted high heterogeneity of studies, total reliance on retrospective studies, and narrow geographical scope as limitations.

Study	Objective(s)	Main finding(s)	Limitation(s)
**Zheng et al. 2020 [42]**	• To find risk factors for the progression of COVID-19 to help reduce the risk of critical illness and death.	• Male, older than 65, and smoking were risk factors for disease progression in patients with COVID-19.<br>• The proportion of underlying diseases such as hypertension, diabetes, cardiovascular disease, and respiratory disease were statistically significant higher in critical/mortal patients compared to the non-critical patients.	• Studies most from China.
**Zuin et al. 2020 [43]**	• Comparison of mortality for COVID-19 patients with and without hypertension.	• Increased mortality when those with hypertension compared to those without, OR 3.36 (CI 1.96-5.74).	• Search conducted up to 23rd March<br>• Only three articles were included.

Table S2. Quality assessment results using Joanna Briggs Institute Critical Appraisal Tools

Study	QA Type	Q1	Q2	Q3	Q4	Q5	Q6	Q7	Q8	Q9	Q10	Q11	Total Y	Total N	Total U	Total NA	QA score
**Alberici, 2020 [44]**	Cohort	Y	Y	Y	Y	Y	Y	Y	Y	Y	NA	Y	10	0	0	1	100
**Al-sabah, 2020 [45]**	Cohort	Y	Y	Y	Y	Y	U	Y	U	N	NA	Y	7	1	2	1	70
**Bianchetti, 2020 [46]**	Cohort	Y	Y	Y	Y	Y	Y	Y	N	U	U	Y	8	1	2	0	73
**Chen, 2020a [47]**	Cohort	Y	Y	Y	Y	Y	Y	Y	Y	N	Y	Y	10	1	0	0	91
**Chen, 2020b [48]**	Cohort	Y	Y	Y	Y	Y	Y	Y	U	U	N	Y	8	1	2	0	73
**Chen, 2020c [49]**	Cohort	Y	Y	Y	Y	Y	N	Y	U	Y	NA	Y	8	1	1	1	80
**Chen, 2020d [50]**	Cohort	Y	Y	Y	N	NA	Y	Y	U	U	NA	Y	6	1	2	2	67
**Chroboczek, 2020 [51]**	Cohort	Y	Y	Y	Y	N	N	Y	U	Y	NA	Y	7	2	1	1	70
**Cummings, 2020 [52]**	Cohort	Y	Y	Y	Y	Y	Y	Y	Y	N	N	Y	9	2	0	0	82
**Docherty, 2020 [53]**	Cohort	Y	Y	Y	N	NA	Y	Y	Y	Y	NA	Y	8	1	0	2	89
**Foy, 2020 [54]**	Cohort	Y	Y	Y	Y	Y	Y	Y	N	Y	Y	Y	10	1	0	0	91
**Gaibazzi, 2020 [55]**	Cohort	Y	Y	Y	Y	Y	Y	Y	U	Y	NA	Y	9	0	1	1	90
**Gao, 2020 [56]**	Cohort	Y	Y	Y	Y	Y	Y	Y	N	Y	NA	Y	9	1	0	1	90
**Giacomelli, 2020 [57]**	Cohort	Y	Y	Y	Y	Y	Y	Y	Y	Y	Y	Y	11	0	0	0	100
**Giorgi-Rossi, 2020 [58]**	Cohort	Y	Y	Y	Y	Y	Y	Y	U	Y	N	Y	9	1	1	0	82
**Huang, 2020 [59]**	Cohort	Y	Y	Y	Y	Y	Y	Y	U	Y	NA	Y	9	0	1	1	90
**Hur, 2020 [60]**	Cohort	Y	Y	Y	Y	Y	Y	Y	U	N	Y	Y	9	1	1	0	82
**Kalligeros, 2020 [61]**	Cohort	Y	Y	Y	Y	Y	Y	Y	U	N	N	Y	8	2	1	0	73
**Kim, 2020 [62]**	Cohort	Y	Y	Y	Y	Y	Y	Y	U	Y	NA	Y	9	0	1	1	90
**Klang, 2020 [63]**	Cohort	Y	Y	Y	Y	Y	Y	Y	U	U	Y	Y	9	0	2	0	82
**Li, 2020 [64]**	Cohort	Y	Y	Y	Y	Y	Y	Y	N	N	N	Y	8	3	0	0	73
**Liu, 2020 [65]**	Cohort	Y	Y	Y	Y	Y	Y	Y	U	U	N	Y	8	1	2	0	73
**Mehta, 2020 [66]**	Cohort	Y	Y	Y	Y	Y	Y	Y	U	N	Y	Y	9	1	1	0	82
**Murillo-Zamora, 2020 [67]**	Case-control	Y	Y	Y	Y	Y	Y	Y	Y	Y	Y	NA	10	0	0	0	91
**Palaiodimos, 2020 [68]**	Cohort	Y	Y	Y	Y	Y	N	Y	Y	N	N	Y	8	3	0	0	73
**Petrilli, 2020 [69]**	Cohort	Y	Y	Y	Y	Y	Y	Y	U	Y	Y	Y	10	0	1	0	91

Study	QA Type	Q1	Q2	Q3	Q4	Q5	Q6	Q7	Q8	Q9	Q10	Q11	Total Y	Total N	Total U	Total NA	QA score
**Regina, 2020 [70]**	Cohort	Y	Y	Y	Y	Y	Y	Y	Y	Y	NA	Y	10	0	0	1	100
**Reyes, 2020 [71]**	Cohort	Y	Y	Y	Y	Y	Y	Y	Y	Y	NA	Y	10	0	0	1	100
**Sapey, 2020 [72]**	Cohort	Y	Y	Y	Y	Y	Y	Y	N	Y	Y	Y	10	1	0	0	91
**Shi, 2020a [73]**	Cohort	Y	Y	Y	Y	Y	Y	Y	N	Y	NA	Y	9	1	0	1	90
**Shi, 2020b [74]**	Case-control	Y	Y	Y	Y	Y	Y	Y	Y	U	Y	NA	9	0	1	0	82
**Shi, 2020c [75]**	Cohort	Y	Y	Y	Y	N	Y	Y	Y	U	U	Y	8	1	2	0	73
**Simonnet, 2020 [76]**	Cohort	Y	Y	Y	Y	Y	Y	Y	N	Y	NA	Y	9	1	0	1	90
**Tang, 2020 [77]**	Cohort	Y	Y	Y	Y	Y	Y	Y	Y	Y	NA	Y	10	0	0	1	100
**Wang, 2020a [78]**	Cohort	Y	Y	Y	Y	Y	Y	Y	N	Y	NA	Y	9	1	0	1	90
**Wang, 2020b [79]**	Case series	Y	Y	Y	Y	Y	Y	Y	Y	Y	Y	NA	10	0	0	0	91
**Wang, 2020c [80]**	Cohort	Y	Y	Y	Y	Y	Y	Y	Y	Y	Y	NA	10	0	0	0	91
**Xie, 2020 [81]**	Cohort	Y	Y	Y	Y	Y	Y	Y	N	Y	NA	Y	9	1	0	1	90
**Zhang, 2020a [82]**	Cohort	Y	Y	Y	Y	Y	Y	Y	Y	Y	Y	Y	11	0	0	0	100
**Zhang, 2020b [83]**	Cohort	Y	Y	Y	Y	Y	Y	Y	U	U	U	Y	8	0	3	0	73

Table S3. Summary of findings from studies not included in the meta-analysis: age and mortality

Study	QA	N	Risk group	Reference	Effect size with 95% CI
**Chen, 2020b [48]**	73	1590	65-74	<65	3.43 (1.24–9.50)
			>75	<65	7.86 (2.44–25.35)
**Liu, 2020 [65]**	73	245	>60	<60	1.09 (1.06–1.13)
**Docherty, 2020 [53]**	89	8341	50-69	<50	4.02 (2.88–5.63)
			70-79	<50	9.59 (6.89–13.34)
			>80	<50	13.59 (9.79–18.85)
**Sapey, 2020 [72]**	91	1663	per z score	—	2.80 (1.92–4.09)
**Giorgi-Rossi, 2020 [58]**	82	42926	50-59	18-49	2.43 (1.95–3.02)
			60-69	18-49	6.60 (5.40–8.06)
			70-79	18-49	17.8 (14.70–21.70)
			80-89	18-49	31.7 (26.10–38.50)
			>90	18-49	52.6 (42.6–65.00)
**Kim, 2020 [62]**	90	2491	40-49	18-39	1.23 (0.51–2.99)
			50-64	18-39	3.11 (1.50–6.46)
			65-74	18-39	5.77 (2.64–12.64)
			75-84	18-39	7.67 (3.35–17.59)
			>85	18-39	10.98 (5.09–23.69)
**Klang, 2020 [63]**	82	3406	<50	>50	3.00 (1.90–4.80)
			>50	<50	1.70 (1.60–1.80)
**Murillo-Zamora, 2020 [67]**	91	5393	30-44	18-29	1.47 (0.96–2.27)
			45-59	18-29	1.99 (1.31–3.02)
			>60	18-29	2.57 (1.69–3.92)
**Petrilli, 2020 [69]**	91	2741	45-54	19-44	2.59 (1.56–4.32)
			55-64	19-44	4.40 (2.73–7.11)
			65-74	19-44	6.99 (4.34–11.27)
			>75	19-44	10.34 (6.37–16.79)
**Shi, 2020b [74]**	82	306	>70	<70	5.87 (1.88–18.33)

QA = quality assessment score; N = number of subjects.

Table S4. Summary of findings from studies not included in the meta-analysis: BMI and mortality

Study	QA	N	Risk group	Reference	Effect size with 95% CI
**Cummings, 2020 [52]**	82	257	>35	<35	0.94 (0.55–1.77)
**Palaiodimos, 2020 [68]**	73	200	<25	25-35	1.37 (0.52–3.64)
			>35	25-35	3.78 (1.45–9.83)
**Petrilli, 2020 [69]**	91	2741	25-30	<25	0.91 (0.74–1.11)
			30-40	<25	1.02 (0.82–1.27)
			>40	<25	1.41 (0.98–2.01)
**Klang, 2020 [63]**	82	3406	30-40	<30	1.10 (0.90–1.30)
			>40	<30	1.60 (1.20–2.30)

QA = quality assessment score; N = number of subjects.

Table S5. Summary of findings from studies not included in the meta-analysis: other risk factors and mortality

Study	QA	N	Risk group	Reference	Effect size with 95% CI
**Sapey, 2020 [72]**	91	1663	Any comorbidities	—	2.15 (1.50–3.09)
**Liu, 2020 [65]**	73	245	Chronic liver diseases	—	2.67 (0.50–14.37)
**Shi, 2020a [73]**	90	416	Renal failure	—	1.10 (0.49–2.44)
**Palaiodimos, 2020 [68]**	73	200	Heart Failure	—	1.43 (0.50–4.06)
**Petrilli, 2020 [69]**	91	2741	Heart Failure	—	1.77 (1.43–2.20)
**Kim, 2020 [62]**	90	2490	Immunocompromised	—	1.39 (1.13–1.70)
**Docherty, 2020 [53]**	89	8341	Malignancy	—	1.19 (1.03–1.38)
**Zhang, 2020b [83]**	73	775	Malignancy	—	1.00 (0.13–7.73)
**Chen, 2020d [50]**	67	904	Neurological disease	—	10.79 (3.62–32.14)
**Kim, 2020 [62]**	90	2490	Neurological disease	—	1.25 (1.04–1.50)
**Docherty, 2020 [53]**	89	8341	Dementia	—	1.39 (1.22–1.58)
**Bianchetti, 2020 [46]**	73	627	Dementia	—	1.84 (1.08–3.13)
**Murillo-Zamora, 2020 [67]**	91	5393	Asthma	—	0.92 (0.68–1.25)
**Petrilli, 2020 [69]**	91	2741	Black	White	0.78 (0.60–1.02)
			Asian	White	1.29 (0.94–1.77)
			Hispanic	White	1.17 (0.95–1.44)
**Kim, 2020 [62]**	90	2490	Black	White	1.07 (0.85–1.35)
			Hispanic	White	1.17 (0.91–1.51)

QA = quality assessment score; N = number of subjects.

Table S6. Summary of findings from studies not included in the meta-analysis: risk factors and ICU admission

Study	QA	N	Risk group	Reference	Effect size with 95% CI
**Kim, 2020 [62]**	90	2491	Age: 40-49	18-39	1.22 (0.96–1.56)
			Age: 50-64	18-39	1.53 (1.28–1.83)
			Age: 65-74	18-39	1.65 (1.34–2.03)
			Age:75-84	18-39	1.84 (1.60–2.11)
			Age: >85	18-39	1.43 (1.00–2.04)
**Wang, 2020b [79]**	91	209	Age: per 1 year	—	0.998 (0.95–1.05)
**Kalligeros, 2020 [61]**	73	103	BMI: 25-30	<25	2.27 (0.59–8.83)
			BMI: 30-35	<25	2.65 (0.64–10.95)
			BMI: >35	<25	5.39 (1.13–25.64)
**Al-sabah, 2020 [45]**	70	1158	BMI: 25-30	<25	1.91 (0.94–3.84)
			BMI: 30-35	<25	2.70 (1.17–6.20)
			BMI: 35-40	<25	1.61 (0.50–5.15)
			BMI: >40	<25	3.95 (1.00–15.20)
**Kim, 2020 [62]**	90	2490	Obesity	Non-obese	1.31 (1.16–1.47)
**Chen, 2020a [47]**	91	249	Any comorbidities	—	1.83 (0.50–6.75)
**Mehta, 2020 [66]**	82	421	ACEi	—	1.77 (1.07–2.92)
**Mehta, 2020 [66]**	82	421	ARB	—	1.16 (0.67–2.02)
**Kim, 2020 [62]**	90	2490	ARB	—	1.07 (0.95–1.21)
**Kim, 2020 [62]**	90	2490	Cardiovascular diseases	—	0.98 (0.88–1.09)
**Shi, 2020b [74]**	82	306	Cardiovascular diseases	—	1.08 (0.25–4.70)
**Shi, 2020c [75]**	73	671	Coronary heart diseases	—	1.63 (0.61–4.40)
**Shi, 2020c [75]**	73	671	Heart failure	—	1.75 (0.52–5.91)
**Kim, 2020 [62]**	90	2490	Immuno-compromised	—	1.29 (1.13–1.47)
**Kim, 2020 [62]**	90	2490	Neurological disease	—	0.85 (0.70–1.04)
**Kim, 2020 [62]**	90	2490	Chronic lung diseases	—	1.17 (1.00–1.37)
**Shi, 2020b [74]**	82	306	Chronic lung diseases	—	1.21 (0.28–5.15)
**Kalligeros, 2020 [61]**	73	103	Black	White	0.80 (0.26–2.45)
			Hispanic	White	0.56 (0.19–1.58)
**Kim, 2020 [62]**	90	2490	Black	White	1.01 (0.89–1.15)
			Hispanic	White	0.96 (0.76–1.21)

QA = quality assessment score; N = number of subjects; ACEi = use of angiotensin-converting-enzyme inhibitors; ARB = use of angiotensin receptor blockers.

Table S7. Summary of findings from studies not included in the meta-analysis: risk factors and invasive mechanical ventilation

Study	QA	N	Risk group	Reference	Effect size with 95% CI
**Hur, 2020 [60]**	82	486	Age: >60	<60	3.90 (2.30–6.76)
**Kalligeros, 2020 [61]**	73	103	BMI: 25-30	<25	3.70 (0.60–22.87)
			BMI: 30-35	<25	6.85 (1.05–44.82)
			BMI: >35	<25	9.99 (1.39–71.69)
**Simonnet, 2020 [76]**	90	124	BMI: 25-30	<25	1.69 (0.52–5.48)
			BMI: 30-35	<25	3.45 (0.83–14.31)
			BMI: >35	<25	7.36 (1.63–33.14)
**Palaiodimos, 2020 [68]**	73	200	BMI: <25	25-35	0.76 (0.26–2.22)
			BMI: >35	25-35	3.87 (1.47–10.18)
**Hur, 2020 [60]**	82	486	BMI: 30-40	<30	1.46 (0.87–2.46)
			BMI: >40	<30	1.92 (0.92–4.00)
**Mehta, 2020 [66]**	82	421	ACEi	—	1.35 (0.74–2.47)
**Mehta, 2020 [66]**	82	421	ARB	—	1.12 (0.59–2.12)
**Palaiodimos, 2020 [68]**	73	200	Hyperlipidaemia	—	1.66 (0.78–3.55)
**Kalligeros, 2020 [61]**	73	103	Black	White	1.83 (0.55–6.11)
			Hispanic	White	1.17 (0.36–3.82)
**Hur, 2020 [60]**	82	486	Black	White	0.56 (0.30–1.01)
			Hispanic	White	0.83 (0.44–1.55)
			Asian	White	0.71 (0.27–1.71)

QA = quality assessment score; N = number of subjects; ACEi = use of angiotensin-converting-enzyme inhibitors; ARB = use of angiotensin receptor blockers.

Figure S1. Forest plots showing meta-analysis results of risk factors for ICU admission

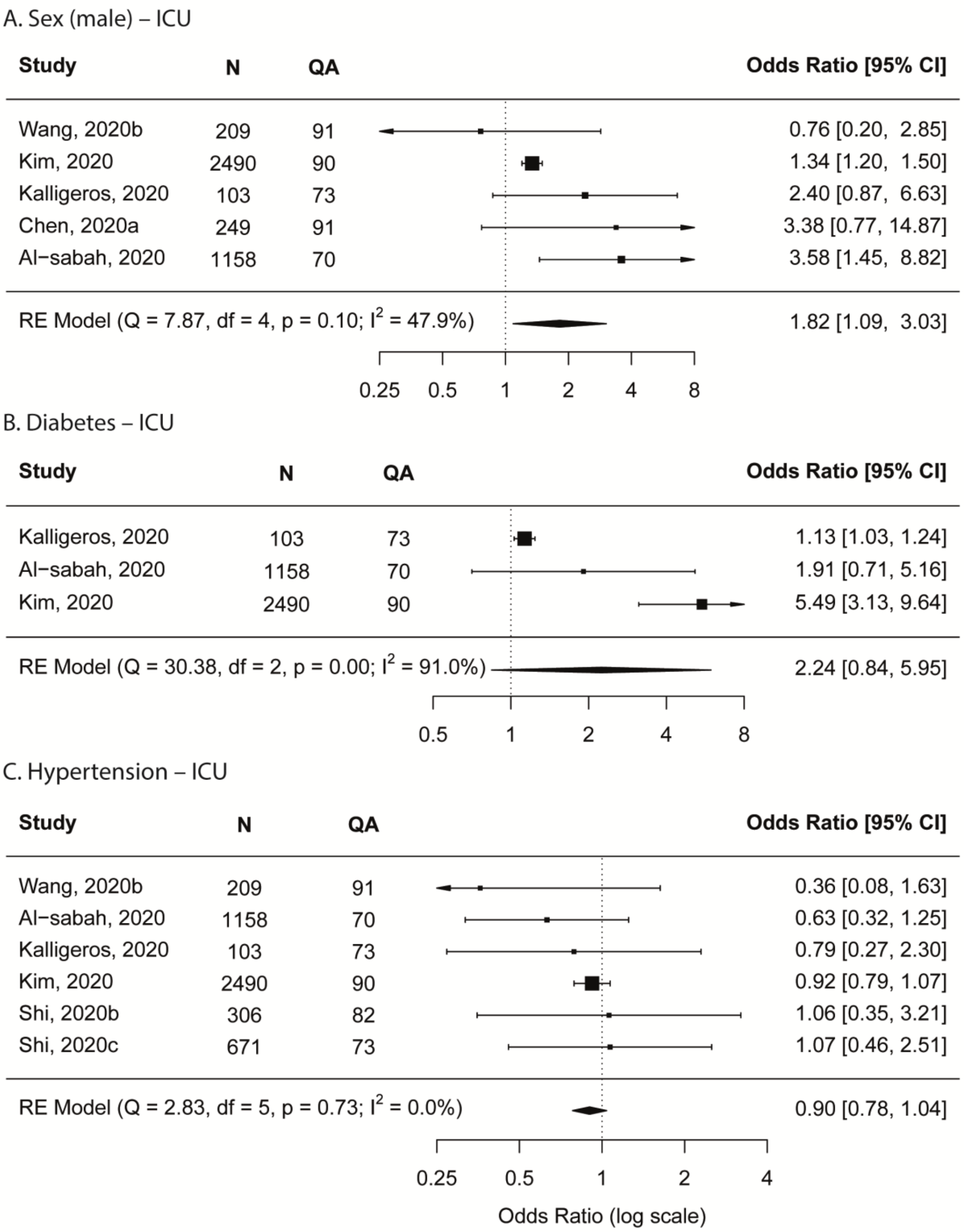

N = number of subjects; QA = quality assessment score.

Figure S2. Forest plots showing meta-analysis results of risk factors for invasive mechanical ventilation

A. Sex (male) – IMV

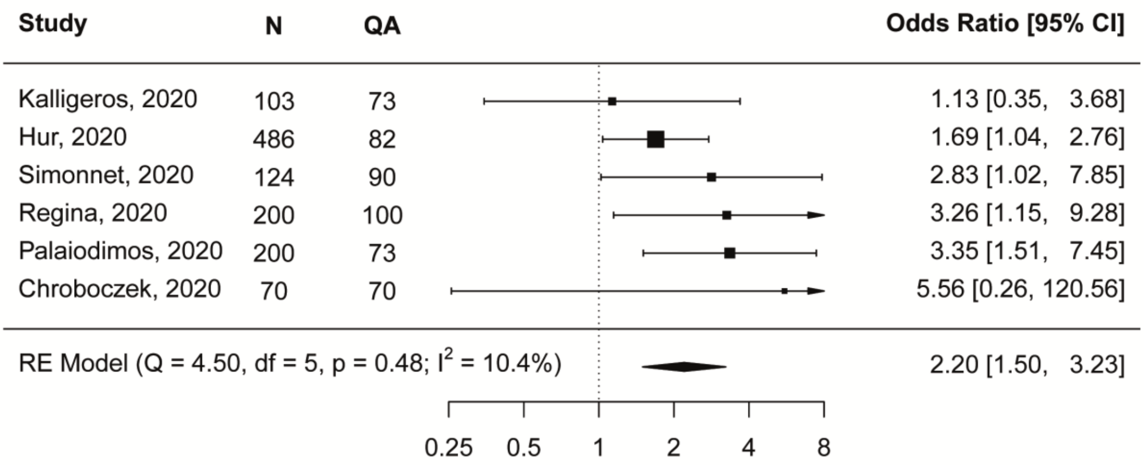

B. Diabetes – IMV

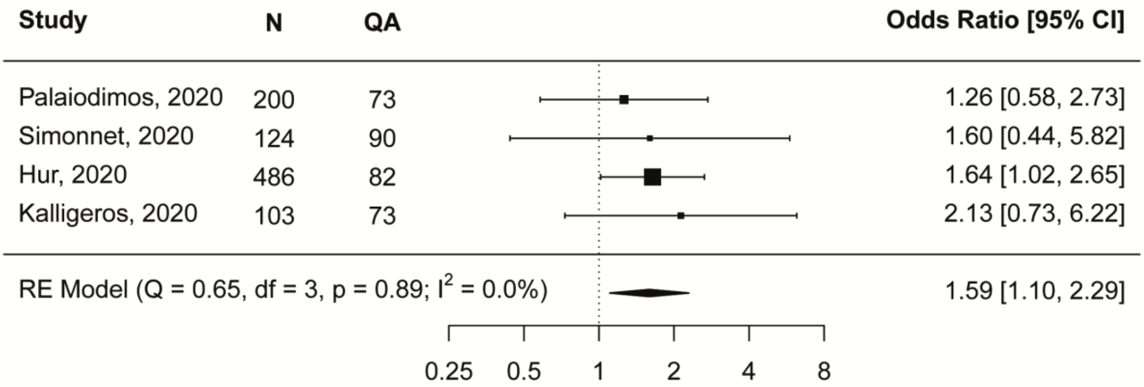

C. Hypertension – IMV

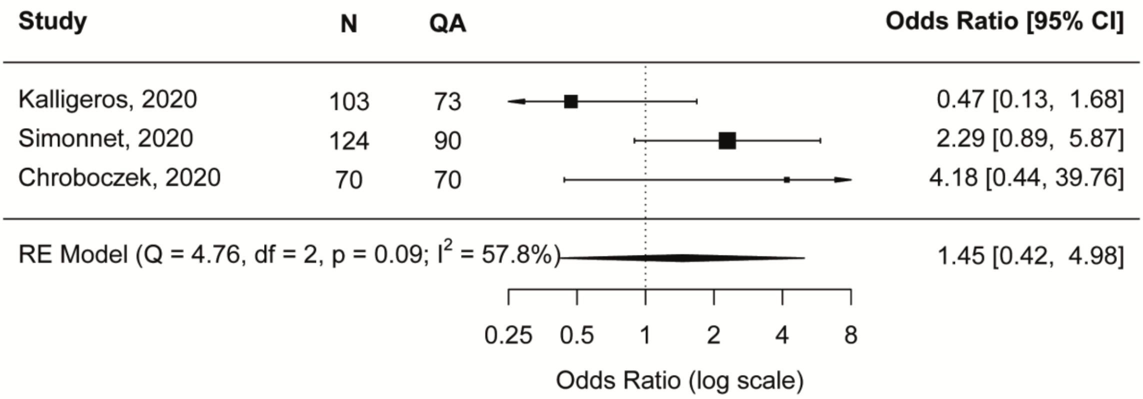

N = number of subjects; QA = quality assessment score.

Reference

1. Shokraneh F. Keeping up with studies on covid-19: systematic search strategies and resources. BMJ. 2020;369:m1601.
2. Aggarwal G, Cheruiyot I, Aggarwal S, Wong J, Lippi G, Lavie CJ, et al. Association of Cardiovascular Disease With Coronavirus Disease 2019 (COVID-19) Severity: A Meta-Analysis. Curr Probl Cardiol. 2020;45(8):100617.
3. Aghagoli G, Gallo Marin B, Soliman LB, Sellke FW. Cardiac involvement in COVID-19 patients: Risk factors, predictors, and complications: A review. J Card Surg. 2020;35(6):1302-5.
4. Alqahtani JS, Oyelade T, Aldhahir AM, Alghamdi SM, Almehmadi M, Alqahtani AS, et al. Prevalence, Severity and Mortality associated with COPD and Smoking in patients with COVID-19: A Rapid Systematic Review and Meta-Analysis. medRxiv. 2020.
5. Baral R, White M, Vassiliou VS. Impact of hospitalised patients with COVID-19 taking Renin-Angiotensin-Aldosterone System inhibitors: a systematic review and meta-analysis. medRxiv. 2020.
6. Bellou V, Tzoulaki I, Evangelou E, Belbasis L. Risk factors for adverse clinical outcomes in patients with COVID-19: A systematic review and meta-analysis. MedRxiv. 2020.
7. Bezabih YM, Bezabih A, Alamneh E, Peterson GM, Bezabhe WM. Comparison of renin-angiotensin-aldosterone system inhibitors with other antihypertensives in association with coronavirus disease-19 clinical outcomes: systematic review and meta-analys. medRxiv. 2020.
8. Bin Abdulhak AA, Kashour T, Noman A, Tlayjeh H, Mohsen A, Al-Mallah MH, et al. Angiotensin Converting Enzyme Inhibitors and Angiotensin Receptor Blockers and Outcome of COVID-19 : A Systematic Review and Meta-analysis. medRxiv. 2020.
9. Gao Y, Liu M, Shi S, Chen Y, Sun Y, Chen J, et al. Cancer is associated with the severity and mortality of patients with COVID-19: a systematic review and meta-analysis. medRxiv. 2020.
10. Ghosal S, Mukherjee JJ, Sinha B, Gangopadhyay KK. The effect of angiotensin converting enzyme inhibitors and angiotensin receptor blockers on death and severity of disease in patients with coronavirus disease 2019 (COVID-19): A meta-analysis. medRxiv. 2020.
11. Grover A, Oberoi M. A systematic review and meta-analysis to evaluate the clinical outcomes in COVID -19 patients on angiotensin converting enzyme inhibitors or angiotensin receptor blockers. MedRxiv. 2020.
12. Hage R, Steinack C, Benden C, Schuurmans MM. COVID-19 in Patients with Solid Organ Transplantation: A Systematic Review. Transplantology. 2020;1(1):1-15.
13. Hessami A, Shamshirian A, Heydari K, Pourali F, Alizadeh-Navaei R, Moosazadeh M, et al. Cardiovascular Diseases and COVID-19 Mortality and Intensive Care Unit Admission: A Systematic Review. medRxiv. 2020.
14. Hu Y, Sun J, Dai Z, Deng H, Li X, Huang Q, et al. Prevalence and severity of corona virus disease 2019 (COVID-19): A systematic review and meta-analysis. J Clin Virol. 2020;127:104371.
15. Huang I, Lim MA, Pranata R. Diabetes mellitus is associated with increased mortality and severity of disease in COVID-19 pneumonia - A systematic review, meta-analysis, and meta-regression. Diabetes Metab Syndr. 2020;14(4):395-403.
16. Huang I, Pranata R. Lymphopenia in severe coronavirus disease-2019 (COVID-19): systematic review and meta-analysis. J Intensive Care. 2020;8:36.
17. Islam MS, Barek MA, Aziz MA, Aka TD, Jakaria M. Association of age, sex, comorbidities, and clinical symptoms with the severity and mortality of COVID-19 cases: a meta-analysis with 85 studies and 67299 cases. medRxiv. 2020.
18. Jain V, Yuan JM. Systematic review and meta-analysis of predictive symptoms and comorbidities for severe COVID-19 infection. MedRxiv. 2020.
19. Jutzeler CR, Bourguignon L, Weis CV, Tong B, Wong C, Rieck B, et al. Comorbidities, clinical signs and symptoms, laboratory findings, imaging features, treatment strategies, and outcomes in adult and pediatric patients with COVID-19: A systematic review and meta-analysis. medRxiv. 2020.

20. Kumar A, Arora A, Sharma P, Anikhindi SA, Bansal N, Singla V, et al. Is diabetes mellitus associated with mortality and severity of COVID-19? A meta-analysis. Diabetes Metab Syndr. 2020;14(4):535-45.
21. Li B, Yang J, Zhao F, Zhi L, Wang X, Liu L, et al. Prevalence and impact of cardiovascular metabolic diseases on COVID-19 in China. Clin Res Cardiol. 2020;109(5):531-8.
22. Liguoro I, Pilotto C, Bonanni M, Ferrari ME, Pusiol A, Nocerino A, et al. SARS-COV-2 infection in children and newborns: a systematic review. Eur J Pediatr. 2020;179(7):1029-46.
23. Lippi G, Wong J, Henry BM. Hypertension in patients with coronavirus disease 2019 (COVID-19): a pooled analysis. Pol Arch Intern Med. 2020;130(4):304-9.
24. Liu X, Long C, Xiong Q, Ma J, Chen C, Su Y, et al. Inflammation Level Severity and Death in Patients With COVID-19: A Rapid Systematic Review and Meta-Analysis. medRxiv. 2020.
25. Ludvigsson JF. Systematic review of COVID-19 in children shows milder cases and a better prognosis than adults. Acta Paediatr. 2020;109(6):1088-95.
26. Martins-Filho PR, Tavares CSS, Santos VS. Factors associated with mortality in patients with COVID-19. A quantitative evidence synthesis of clinical and laboratory data. Eur J Intern Med. 2020;76:97-9.
27. Mason KE, McHale P, Pennington A, Maudsley G, Day J, Barr B. Age-adjusted associations between comorbidity and outcomes of COVID-19: a review of the evidence. medRxiv. 2020.
28. Matsushita K, Ding N, Kou M, Hu X, Chen M, Gao Y, et al. The relationship of COVID-19 severity with cardiovascular disease and its traditional risk factors: A systematic review and meta-analysis. MedRxiv. 2020.
29. Nasiri MJ, Haddadi S, Tahvildari A, Farsi Y, Arbabi M, Hasanzadeh S, et al. COVID-19 clinical characteristics, and sex-specific risk of mortality: Systematic Review and Meta-analysis. medRxiv. 2020.
30. Parohan M, Yaghoubi S, Seraji A, Javanbakht MH, Sarraf P, Djalali M. Risk factors for mortality of adult inpatients with Coronavirus disease 2019 (COVID-19): a systematic review and meta-analysis of retrospective studies. MedRxiv. 2020.
31. Parohan M, Yaghoubi S, Seraji A. Liver injury is associated with severe coronavirus disease 2019 (COVID-19) infection: A systematic review and meta-analysis of retrospective studies. Hepatol Res. 2020;50(8):924-35.
32. Pigoga JL, Friedman A, Broccoli M, Hirner S, Naidoo AV, Singh S, et al. Clinical and historical features associated with severe COVID-19 infection: a systematic review. medRxiv. 2020.
33. Pranata R, Huang I, Lim MA, Wahjoepramono EJ, July J. Impact of cerebrovascular and cardiovascular diseases on mortality and severity of COVID-19–systematic review, meta-analysis, and meta-regression. Journal of Stroke and Cerebrovascular Diseases. 2020;29(8).
34. Pranata R, Huang I, Lukito AA, Raharjo SB. Elevated N-terminal pro-brain natriuretic peptide is associated with increased mortality in patients with COVID-19: systematic review and meta-analysis. Postgrad Med J. 2020;96(1137):387-91.
35. Pranata R, Lim MA, Huang I, Raharjo SB, Lukito AA. Hypertension is associated with increased mortality and severity of disease in COVID-19 pneumonia: A systematic review, meta-analysis and meta-regression. J Renin Angiotensin Aldosterone Syst. 2020;21(2):1470320320926899.
36. Roncon L, Zuin M, Rigatelli G, Zuliani G. Diabetic patients with COVID-19 infection are at higher risk of ICU admission and poor short-term outcome. J Clin Virol. 2020;127:104354.
37. Ssentongo P, Ssentongo AE, Heilbrunn ES, Ba DM, Chinchilli VM. The association of cardiovascular disease and other pre-existing comorbidities with COVID-19 mortality: A systematic review and meta-analysis. MedRxiv. 2020.
38. Tamara A, Tahapary DL. Obesity as a predictor for a poor prognosis of COVID-19: A systematic review. Diabetes Metab Syndr. 2020;14(4):655-9.
39. Tian W, Jiang W, Yao J, Nicholson CJ, Li RH, Sigurslid HH, et al. Predictors of mortality in hospitalized COVID-19 patients: A systematic review and meta-analysis. J Med Virol. 2020.

40. Vardavas CI, Nikitara K. COVID-19 and smoking: A systematic review of the evidence. Tob Induc Dis. 2020;18:20.
41. Zhao X, Zhang B, Li P, Ma C, Gu J, Hou P, et al. Incidence, clinical characteristics and prognostic factor of patients with COVID-19: a systematic review and meta-analysis medRxiv. 2020.
42. Zheng Z, Peng F, Xu B, Zhao J, Liu H, Peng J, et al. Risk factors of critical & mortal COVID-19 cases: A systematic literature review and meta-analysis. J Infect. 2020;81(2):e16-e25.
43. Zuin M, Rigatelli G, Zuliani G, Rigatelli A, Mazza A, Roncon L. Arterial hypertension and risk of death in patients with COVID-19 infection: Systematic review and meta-analysis. J Infect. 2020;81(1):e84-e6.
44. Alberici F, Delbarba E, Manenti C, Econimo L, Valerio F, Pola A, et al. A report from the Brescia Renal COVID Task Force on the clinical characteristics and short-term outcome of hemodialysis patients with SARS-CoV-2 infection. Kidney Int. 2020;98(1):20-6.
45. Al-Sabah SK, Al-Haddad M, Al Youha S, Jamal MH, Almazeedi S. COVID-19: Impact of Obesity and Diabetes in Disease Severity. medRxiv2020.
46. Bianchetti A, Rozzini R, Guerini F, Boffelli S, Ranieri P, Minelli G, et al. Clinical Presentation of COVID19 in Dementia Patients. J Nutr Health Aging. 2020;24(6):1-3.
47. Chen J, Qi T, Liu L, Ling Y, Qian Z, Li T, et al. Clinical progression of patients with COVID-19 in Shanghai, China. J Infect. 2020;80(5):e1-e6.
48. Chen R, Liang W, Jiang M, Guan W, Zhan C, Wang T, et al. Risk Factors of Fatal Outcome in Hospitalized Subjects With Coronavirus Disease 2019 From a Nationwide Analysis in China. Chest. 2020;158(1):97-105.
49. Chen C, Yi ZJ, Chang L, Shuo HZ, Ming Z, Pei T, et al. The characteristics and death risk factors of 132 COVID-19 pneumonia patients with comorbidities: a retrospective single center analysis in Wuhan, China. medRxiv2020.
50. Chen Y, Yang D, Cheng B, Chen J, Peng A, Yang C, et al. Clinical Characteristics and Outcomes of Patients With Diabetes and COVID-19 in Association With Glucose-Lowering Medication. Diabetes Care. 2020;43(7):1399-407.
51. Chroboczek T, Lacoste M, Wackenheim C, Challan-Belval T, Amar B, Boisson T, et al. Beneficial effect of corticosteroids in severe COVID-19 pneumonia: a propensity score matching analysis. medRxiv2020.
52. Cummings MJ, Baldwin MR, Abrams D, Jacobson SD, Meyer BJ, Balough EM, et al. Epidemiology, clinical course, and outcomes of critically ill adults with COVID-19 in New York City: a prospective cohort study. medRxiv. 2020/06/09 ed2020.
53. Docherty AB, Harrison EM, Green CA, Hardwick HE, Pius R, Norman L, et al. Features of 16,749 hospitalised UK patients with COVID-19 using the ISARIC WHO Clinical Characterisation Protocol. medRxiv2020.
54. Foy BH, Carlson JC, Reinertsen E, Padros Valls R, Pallares Lopez R, Palanques-Tost E, et al. Elevated RDW is Associated with Increased Mortality Risk in COVID-19. medRxiv2020.
55. Gaibazzi N, Martini C, Mattioli M, Tuttolomondo D, Guidorossi A, Suma S, et al. Lung disease severity, Coronary Artery Calcium, Coronary inflammation and Mortality in Coronavirus Disease 2019. medRxiv2020.
56. Gao L, Jiang D, Wen XS, Cheng XC, Sun M, He B, et al. Prognostic value of NT-proBNP in patients with severe COVID-19. Respir Res. 2020;21(1):83.
57. Giacomelli A, Ridolfo AL, Milazzo L, Oreni L, Bernacchia D, Siano M, et al. 30-day mortality in patients hospitalized with COVID-19 during the first wave of the Italian epidemic: a prospective cohort study. Pharmacol Res. 2020;158:104931.
58. Giorgi Rossi P, Ferroni E, Spila Alegiani S, Leoni O, Pitter G, Cereda D, et al. Survival of hospitalized COVID-19 patients in Northern Italy: a population-based cohort study by the ITA-COVID19 Network. medRxiv2020.
59. Huang J, Cheng A, Kumar R, Fang Y, Chen G, Zhu Y, et al. Hypoalbuminemia predicts the outcome of COVID-19 independent of age and co-morbidity. J Med Virol. 2020.

60. Hur K, Price CPE, Gray EL, Gulati RK, Maksimoski M, Racette SD, et al. Factors Associated With Intubation and Prolonged Intubation in Hospitalized Patients With COVID-19. Otolaryngol Head Neck Surg. 2020;163(1):194599820929640.
61. Kalligeros M, Shehadeh F, Mylona EK, Benitez G, Beckwith CG, Chan PA, et al. Association of Obesity with Disease Severity among Patients with COVID-19. Obesity (Silver Spring). 2020;28(7):1200-4.
62. Kim L, Garg S, O'Halloran A, Whitaker M, Pham H, Anderson EJ, et al. Interim Analysis of Risk Factors for Severe Outcomes among a Cohort of Hospitalized Adults Identified through the U.S. Coronavirus Disease 2019 (COVID-19)-Associated Hospitalization Surveillance Network (COVID-NET). medRxiv2020.
63. Klang E, Kassim G, Soffer S, Freeman R, Levin MA, Reich DL. Morbid Obesity as an Independent Risk Factor for COVID-19 Mortality in Hospitalized Patients Younger than 50. Obesity (Silver Spring). 2020.
64. Li K, Chen D, Chen S, Feng Y, Chang C, Wang Z, et al. Radiographic Findings and other Predictors in Adults with Covid-19. medRxiv2020.
65. Liu Y, Du X, Chen J, Jin Y, Peng L, Wang HHX, et al. Neutrophil-to-lymphocyte ratio as an independent risk factor for mortality in hospitalized patients with COVID-19. J Infect. 2020;81(1):e6-e12.
66. Mehta N, Kalra A, Nowacki AS, Anjewierden S, Han Z, Bhat P, et al. Association of Use of Angiotensin-Converting Enzyme Inhibitors and Angiotensin II Receptor Blockers With Testing Positive for Coronavirus Disease 2019 (COVID-19). JAMA Cardiol. 2020.
67. Murillo-Zamora E, Hernandez-Suarez CM. Survival in adult inpatients with COVID-19. medRxiv2020.
68. Palaiodimos L, Kokkinidis DG, Li W, Karamanis D, Ognibene J, Arora S, et al. Severe obesity is associated with higher in-hospital mortality in a cohort of patientswith COVID-19 in the Bronx, New York. medRxiv2020.
69. Petrilli CM, Jones SA, Yang J, Rajagopalan H, O'Donnell L, Chernyak Y, et al. Factors associated with hospital admission and critical illness among 5279 people with coronavirus disease 2019 in New York City: prospective cohort study. Bmj. 2020;369:m1966.
70. Regina J, Papadimitriou-Olivgeris M, Burger R, Filippidis P, Tschopp J, Desgranges F, et al. Epidemiology, risk factors and clinical course of SARS-CoV-2 infected patients in a Swiss university hospital: an observational retrospective study. medRxiv2020.
71. Reyes Gil M, Gonzalez-Lugo JD, Rahman S, Barouqa M, Szymnaski J, Ikemura K, et al. Correlation of coagulation parameters with clinical outcomes in Coronavirus-19 affected minorities in United States: Observational cohort. medRxiv2020.
72. Sapey E, Gallier S, Mainey C, Nightingale P, McNulty D, Crothers H, et al. Ethnicity and risk of death in patients hospitalised for COVID-19 infection: an observational cohort study in an urban catchment area. medRxiv2020.
73. Shi S, Qin M, Shen B, Cai Y, Liu T, Yang F, et al. Association of Cardiac Injury With Mortality in Hospitalized Patients With COVID-19 in Wuhan, China. JAMA Cardiol. 2020.
74. Shi Q, Zhang X, Jiang F, Zhang X, Hu N, Bimu C, et al. Clinical Characteristics and Risk Factors for Mortality of COVID-19 Patients With Diabetes in Wuhan, China: A Two-Center, Retrospective Study. Diabetes Care. 2020.
75. Shi S, Qin M, Cai Y, Liu T, Shen B, Yang F, et al. Characteristics and clinical significance of myocardial injury in patients with severe coronavirus disease 2019. Eur Heart J. 2020.
76. Simonnet A, Chetboun M, Poissy J, Raverdy V, Noulette J, Duhamel A, et al. High prevalence of obesity in severe acute respiratory syndrome coronavirus-2 (SARS-CoV-2) requiring invasive mechanical ventilation. Obesity (Silver Spring). 2020;28(7):1195-9.
77. Tang N, Bai H, Chen X, Gong J, Li D, Sun Z. Anticoagulant treatment is associated with decreased mortality in severe coronavirus disease 2019 patients with coagulopathy. J Thromb Haemost. 2020;18(5):1094-9.

78. Wang D, Yin Y, Hu C, Liu X, Zhang X, Zhou S, et al. Clinical course and outcome of 107 patients infected with the novel coronavirus, SARS-CoV-2, discharged from two hospitals in Wuhan, China. Crit Care. 2020;24(1):188.
79. Wang G, Wu C, Zhang Q, Wu F, Yu B, Lv J, et al. C-Reactive Protein Level May Predict the Risk of COVID-19 Aggravation. Open Forum Infect Dis. 2020;7(5):ofaa153.
80. Wang L, He W, Yu X, Hu D, Bao M, Liu H, et al. Coronavirus disease 2019 in elderly patients: Characteristics and prognostic factors based on 4-week follow-up. J Infect. 2020;80(6):639-45.
81. Xie J, Covassin N, Fan Z, Singh P, Gao W, Li G, et al. Association Between Hypoxemia and Mortality in Patients With COVID-19. Mayo Clin Proc. 2020;95(6):1138-47.
82. Zhang Y, Cui Y, Shen M, Zhang J, Liu B, Dai M, et al. Comorbid Diabetes Mellitus was Associated with Poorer Prognosis in Patients with COVID-19: A Retrospective Cohort Study. medRxiv2020.
83. Zhang H, Shi T, Wu X, Zhang X, Wang K, Bean D, et al. Risk prediction for poor outcome and death in hospital in-patients with COVID-19: derivation in Wuhan, China and external validation in London, UK. medRxiv2020.
